# Structure Prediction of Organic/Inorganic Interfaces with Genarris

**DOI:** 10.1021/acs.jctc.6c00235

**Published:** 2026-04-23

**Authors:** Haoran Ni, Kevin Larkin, Wen Wen, Saeed Moayedpour, Rithwik Tom, Imanuel Bier, Derek Dardzinski, Noa Marom

**Affiliations:** † Department of Materials Science and Engineering, 6612 Carnegie Mellon University, Pittsburgh, Pennsylvania 15213, United States; ‡ Department of Chemistry, 6612 Carnegie Mellon University, Pittsburgh, Pennsylvania 15213, United States; § Department of Physics, 7198 Carnegie Mellon University, Pittsburgh, Pennsylvania 15213, United States

## Abstract

Organic/inorganic interfaces perform critical functions in organic electronic devices, and their structure can affect device performance. In addition, interactions with the substrate can promote the growth of otherwise metastable thin-film structures by epitaxial templating. Predicting the structure of organic/inorganic interfaces by computer simulations can aid in the design of interfaces with desirable properties. We present Genarris Interfaces, a structure generator for organic/inorganic interfaces. Genarris Interfaces uses epitaxy matrices to impose commensurism with the substrate. Film structures are generated in all layer groups that are compatible with the substrate symmetry and the requested number of molecules per unit cell. Clustering and down-selection are performed to reduce the number of structures while maintaining the diversity of packing motifs. Finally, relaxation and stability ranking are performed using dispersion-inclusive density functional theory (DFT). We demonstrate the application of Genarris for the interfaces of PTCDA on Ag(111), TCNE on Au(111), and naphthalene on Cu(111). In all cases, we find generated structures that resemble experimental scanning tunneling microscopy (STM) images. The electronic structure agrees well with spectroscopy experiments, where available. We envision Genarris Interfaces being used to predict the structure of organic/inorganic interfaces, to generate initial populations for other structure prediction algorithms, and to generate data sets for training machine learning models.

## Graphical abstract

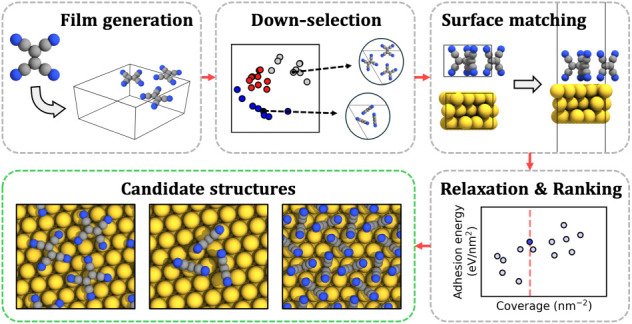

## 1 Introduction

Organic electronic devices offer unique advantages such as flexibility, transparency, and low-cost fabrication.^1–5^ This makes organic electronic devices appealing for low-cost and/or large area applications, as well as wearable devices.^6–8^ In organic electronic devices, such as organic light emitting diodes (OLEDs),^9–11^ organic photovoltaics (OPV)^12,13^ organic photodetectors,^14,15^ and organic field effect transistors (OFETs),^16–20^ current flows to/from external circuits through interfaces between inorganic electrodes and organic active layers.^18,21,22^ Desirable properties for reducing transport losses in devices, such as low injection barrier^23,24^ low contact resistance^24,25^ and few interfacial trap states^26^ are determined by the interface structure.

When an organic semiconductor is brought into contact with an electrode, the electrode Fermi level and the organic transport level, e.g., highest occupied molecular orbital (HOMO) for hole injection and lowest unoccupied molecular orbital (LUMO) for electron injection, establish a specific band alignment, which can be affected the presence of interface dipoles, interface states, and charge transfer.^27–37^ The injection barrier, which largely governs the contact resistance in organic devices, is then set by the energy difference between the electrode Fermi level and the organic transport level.^38^ Structural disorder at the interface can introduce trap states that capture charge carriers, leading to additional losses, increased contact resistance, and reduced carrier mobility.^39,40^ Beyond the effect of organic/inorganic interfaces on device functionality and efficiency, the interaction of organic molecules with a substrate can promote the formation of thin-film polymorphs with a different crystal structure than the common bulk structure of a given compound.^41–43^ This phenomenon, known as epitaxial templating,^44–52^ can be harnessed to promote the growth of phases with desirable properties.^53–59^ Understanding and controlling the structure and properties of organic/inorganic interfaces is therefore of paramount importance for high-performance organic electronic devices.

The structure of organic/inorganic interfaces is often characterized by scanning tunneling microscopy (STM),^60–68^ and their electronic structure is often probed by ultraviolet photoelectron spectroscopy (UPS).^69–72^ Computer simulations can help interpret experimental data by assigning specific atomistic configurations to observed STM images and by assigning UPS peaks to specific states and/or structural features. Moreover, computer simulations can inform the design of interfaces with desired properties. Interfaces comprising different materials can be constructed and the resulting electronic properties can be systematically analyzed in silico, using first-principles simulations.^30,31,73–80^ Thus, simulations could be utilized to screen candidate interfaces and guide experimental studies in the most promising directions.

While computational high-throughput screening has become routine for bulk crystals and isolated molecules, this is not the case for interfaces in general and molecular interfaces in particular, owing to their higher degree of complexity. The vast number of possible molecular configurations on a surface, combined with the large size of interface models, make structure prediction by exhaustive sampling of the high-dimensional configuration space challenging and computationally expensive. Some progress has been made toward developing computational methods to efficiently sample the configuration space of organic/inorganic interfaces. The SAMPLE code,^81–83^ constructs interface structures by combining building blocks that comprise a single molecule adsorbed on a slice of substrate surface in various ways. Machine learning is used to coarse-grain the potential energy landscape and ab initio calculations are only performed on a small subset of interface configurations. The GAMMA code^84^ describes the self-assembly of molecular monolayers on metal surfaces in the low-coverage regime. Similar to SAMPLE, GAMMA assembles structures out of building blocks comprising a molecule on the surface, and uses machine learning to coarse-grain the potential energy landscape. GAMMA also considers entropic contributions and allows for the formation of large-scale nonperiodic structures. The Ogre code^85^ predicts the structure of organic epitaxial interfaces by searching for stable commensurate domains between the bulk crystal structures of the two constituent materials.

Here, we present Genarris Interfaces, an open-source package for structure prediction of molecular interfaces, derived from the Genarris,^86–88^ code for crystal structure prediction. In contrast to SAMPLE and GAMMA, Genarris Interfaces does not make any assumptions regarding the molecule’s adsorption mode on the surface. Unlike Ogre, Genarris Interfaces does not assume that the film adopts its bulk crystal structure. Moreover, Genarris Interfaces makes no assumptions regarding the strength of the interactions between the molecules and the substrate or the intermolecular interactions. Genarris Interfaces only assumes that the molecular film is periodic and commensurate with the substrate surface. For molecular crystal structure prediction, Genarris generates random structures in all the space groups compatible with the molecular symmetry and number of molecules per unit cell.^86–88^ Genarris Interfaces employs a similar strategy to explore the configuration space by generating film structures in all the layer groups^89,90^ that are compatible with the surface unit cell symmetry and the number of molecules per cell. This enables Genarris to generate interface structures with diverse adsorption configurations and packing motifs, with varying surface coverage ranging from submonolayer to multilayer films. Similar to the workflow of Genarris for molecular crystals,^89,90^ Genarris Interfaces performs a sequence of duplicate removal, clustering, and selection steps to reduce the number of structures considered further. Similar to Ogre^85^ Genarris Interfaces performs “surface matching” to preoptimize the position of the generated film on top of the surface using a computationally efficient model. In the final step, the remaining structures are fully relaxed using dispersion-inclusive density functional theory (DFT) and ranked based on adhesion energy. Below, we present a detailed description of Genarris Interfaces followed by three case studies: PTCDA/Ag(111), TCNE/Au(111), and naphthalene/Cu(111). These are representative examples of interfaces of commonly used types of organic semiconductors with coinage metals, for which STM images and spectroscopy data are available. In all cases, Genarris generates interface structures that closely resemble experimental STM images and the resulting electronic structure is in agreement with available spectroscopy data.

## 2 Code Description

### 2.1 Workflow Overview

Genarris Interfaces is written in Python and the film generation algorithm is written in C for efficient large scale generation.^87^ The Ogre^85,91^ code for surface generation and interface structure prediction is called by Genarris Interfaces to perform certain operations, as detailed below. For energy evaluations and geometry relaxations, Genarris Interfaces calls the electronic structure package FHI-aims.^92^ All versions of Genarris and Ogre are available for download from www.noamarom.com under a BSD-3 license.

The workflow of structure prediction of organic/inorganic interfaces is illustrated in Figure 1. The inputs to Genarris Interfaces are the structure files of the organic molecule and inorganic substrate, along with a configuration file for runtime settings. The workflow starts with generation of film structures. A machine-learned model^93^ (also used by Genarris for molecular crystals) is used to estimate the target unit cell volume. Epitaxy matrices are used to impose commensurism between the film and substrate supercells in the plane of the interface. Structures are generated in all the layer groups^89,90^ that are compatible with the symmetry of commensurate substrate supercells, the symmetry of the molecule, and the requested number of molecules per cell. The structures undergo proximity checks to ensure that no two molecules are too close to each other.^86–88^ Structure generation continues until a target number of structures is reached. We note that the PyXtal code^94^ is also able to generate structures in layer groups, however the structures are generated one at a time in a user-specified unit cell and symmetry group and without consideration of a substrate. Thus, Genarris Interfaces is able to perform a more thorough, automated exploration of the configuration space of possible commensurate molecular films on a given substrate.

**Figure 1:**
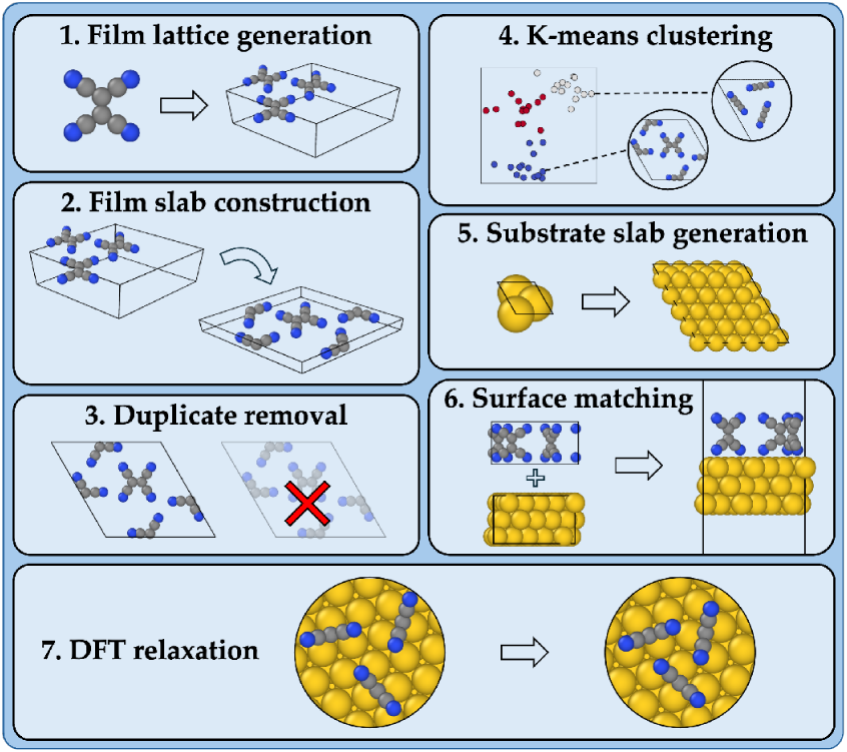
Overall workflow of interface structure generation with Genarris Interfaces.

Subsequently, Ogre is used to turn the generated film unit cells into surface slab models, whose lattice vectors in the plane of the interface are commensurate with the substrate supercell. Ogre is able to generate surface slab models of molecular crystals while keeping the molecules intact.^91^ In addition, Ogre can identify all unique surface terminations. The surface slab models constitute the “raw pool” of film structures. Next, duplicate removal is performed to leave only unique film structures. The remaining structures are sorted by coverage and clustered by structural similarity using *k*-means clustering. Diversity-based selection is performed, after which representative structures of each coverage proceed to interface construction.

Unlike the SAMPLE^81^ and GAMMA^84,95^ codes, Genarris Interfaces makes no assumptions on the molecule’s binding site to the surface or the molecule’s orientation. It can generate structures with low, medium, and high coverage, including multilayer structures. To construct the interface, a commensurate substrate slab is generated for each film slab using the Atomic Simulation Environment (ASE).^96^ Then, surface matching is performed to find the optimal distance and in-plane registry between the substrate and film. Bayesian optimization^97^ (BO) is performed to efficiently search the 3D space of shifts of the film with respect to the substrate in the *x*, *y*, and *z* directions. To avoid performing a large number of expensive DFT calculations for surface matching, we have formulated a geometric score function^98^ designed for organic/inorganic interfaces. The score function serves as the BO objective function. The final pool of surface-matched interface structures proceeds to geometry optimization and ranking with DFT.

### 2.2 Film Unit Cell Generation

Figure 2 shows the workflow of film unit cell generation. Similar to the workflow of Genarris^87,88^ for molecular crystal structure generation, the film structure generation begins with estimating the target unit cell volume. A machine-learned model, based on the molecule’s packing accessible area and the atomic bonding environments present in the molecule (molecular topological fragments), is used to estimate the molecular solid form volume, i.e., the effective volume occupied by a molecule in the unit cell.^93^ The target volume of the film unit cell is then computed based on the number of molecules in the cell (Z). An additional requirement for successful film structure generation is an estimate of the interface area. One half of the estimated volume is used as the maximum allowed interface area. The user may also specify a value for the target interface area if, for example, experimental data is available.

**Figure 2:**
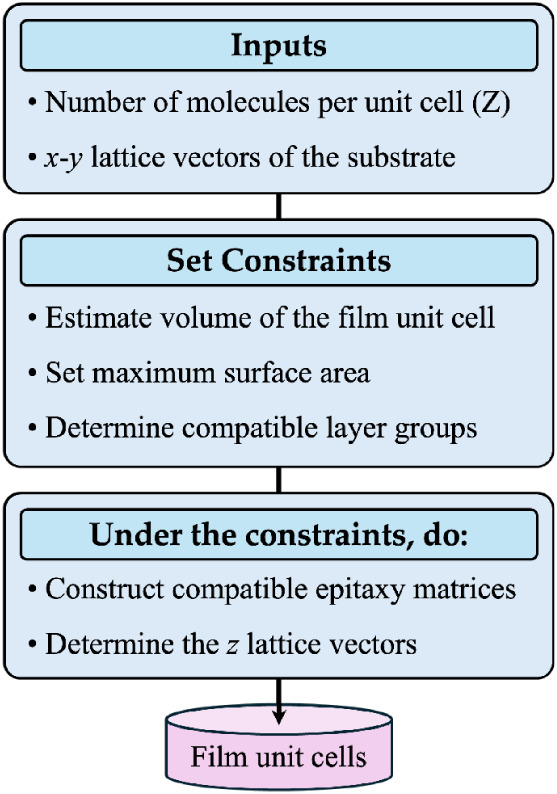
Workflow of film unit cell generation.

Here, we assume that the interactions between the substrate and film are sufficiently strong to impose a commensurate relationship. In addition, commensurability is necessary for performing DFT simulations of interfaces with periodic boundary conditions. For these reasons, others have also focused on commensurate structures.^81,84,95^ We note that in some cases, if the interactions between the substrate and adsorbate are weak, a noncommensurate film may grow (e.g., DM-PBDCI on Ag(111)^99^). This scenario is not covered by Genarris Interfaces.

The 2D substrate unit cell lattice vectors, (*s*_1_, *s*_2_), are obtained from user input. To generate commensurate film structures, the film lattice vectors, (*f*_1_, *f*_2_), are obtained by multiplying the substrate unit cell lattice vectors by an epitaxy matrix, ***E***:

1 $$\left(\begin{array}{c} f_{1}\\ f_{2} \end{array}\right)=E\left(\begin{array}{c} s_{1}\\ s_{2} \end{array}\right)=\left[\begin{array}{cc} E_{1} & E_{2}\\ E_{1} & E_{2} \end{array}\right]\left(\begin{array}{c} s_{1}\\ s_{2} \end{array}\right)$$

In the case of commensurate epitaxy, all elements in the epitaxy matrix are integer numbers.^100^ With this definition, all 2D crystal systems (i.e., oblique, rectangular, square, and hexagonal) that are commensurate with the substrate symmetry can be generated. Genarris Interfaces generates film unit cells with all epitaxy matrices that produce an area below the maximum interface area.

Next, the compatible layer groups and the associated 3D crystal systems are determined based on *Z*, molecular symmetry, and the 2D lattice vectors obtained in the previous step. The symmetries of all film structures can be described by the 80 layer groups.^89^ Unlike space groups, which represent the symmetries of three-dimensional lattices, layer groups represent the symmetry of lattices that are periodic in two dimensions and have a finite thickness in the third dimension. Based on the two-dimensional Bravais system of the periodic interface and the corresponding three-dimensional crystal systems, the layer groups are classified as oblique/triclinic, oblique/monoclinic, rectangle/monoclinic, rectangle/orthorhombic, hexagonal/trigonal, hexagonal/hexagonal and square/tetragonal. Finally, the third lattice vector, in the out-of-plane direction, is generated based on the crystal system corresponding to the layer group and the 2D lattice vectors in the plane of the interface. The resulting film unit cell has a volume *N* times bigger than the estimated volume. The integer *N* is used to accommodate all possible molecular orientations from lying flat on the surface to perpendicular. In addition, it accounts for the fact that film structures are not necessarily close-packed like crystal structures, and sparse films with submonolayer coverage are also possible. A default value of *N* = 3 has been found empirically to produce reasonable structures with diverse molecular orientations.

### 2.3 Molecule Placement

After the film unit cell is generated, molecules are placed in the cell following the same procedure implemented in Genarris for molecular crystals,^87,88^ but using layer group symmetries instead of space groups. The steps are displayed in Figure 3. If the molecule occupies a general Wyckoff position, it is placed randomly inside the unit cell with a random orientation. The layer group symmetry operations are then applied to generate the remaining molecules in the unit cell. Special Wyckoff positions, with the exception of inversion centers, require alignment of the molecule with the site symmetry. This is performed by checking all possible orientations of the molecule with respect to the symmetry directions of the layer group. Genarris constructs a list of all possible molecular axes that may be associated with a symmetry element. The compatibility of molecule placement at a special position is checked against the specified number of molecules per cell. The molecule’s center of mass is placed in the special position, such that one of the molecular axes is oriented along one of the symmetry directions of the layer group. Then, the symmetry operations of the layer group are applied. If the number of molecules that coalesce into one molecule is equal to the order of the site, the special Wyckoff position is regarded as compatible. If not, different molecular axes and symmetry directions are considered until all possible combinations are exhausted. Depending on the site symmetry of the special position, the allowed degrees of freedom are randomized. For instance, a molecule with a 2-fold axis of rotation can placed at a suitable Wyckoff position provided the molecular axis coincides with the 2-fold site symmetry axis. The molecule is free to rotate about this axis while still satisfying the site symmetry of the Wyckoff position. Once a molecule is successfully placed in a special position, the layer group symmetry operations are used to generate the remaining molecules in the cell.

**Figure 3:**
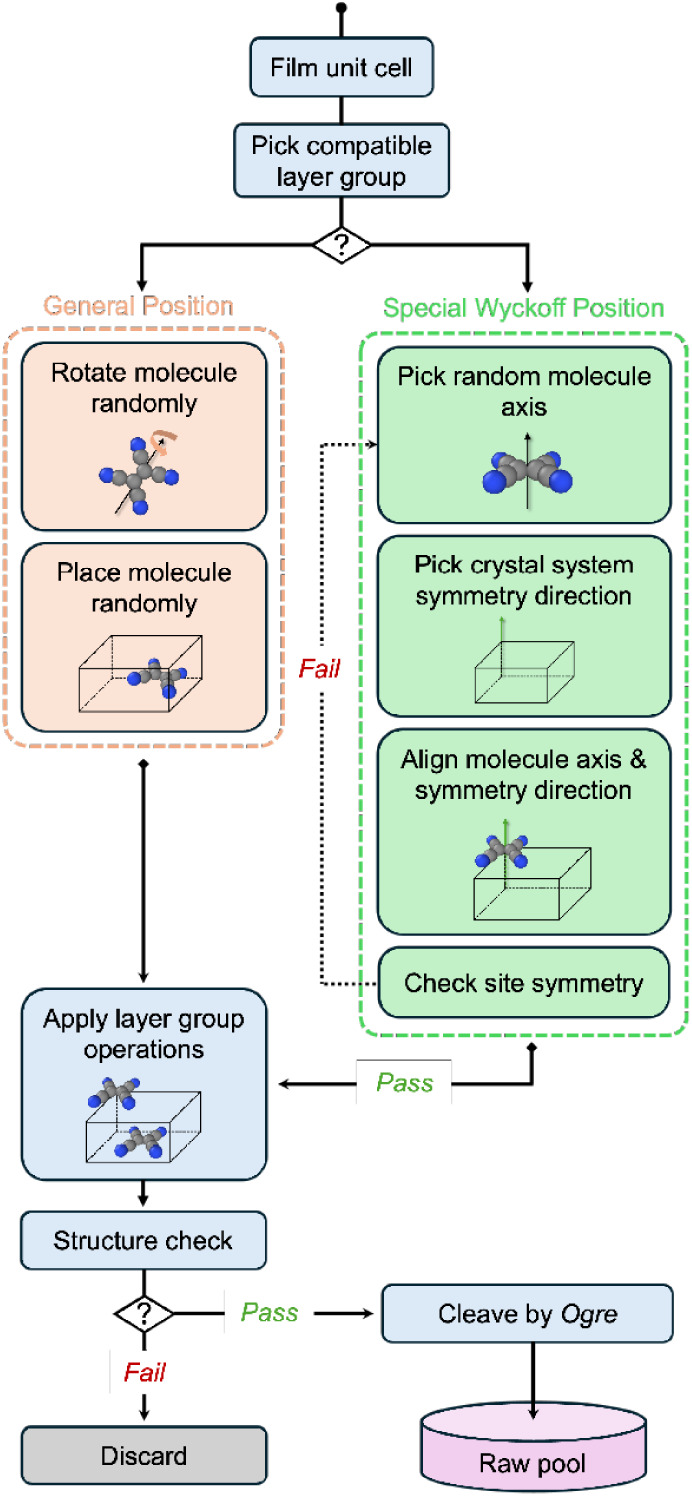
Workflow of film molecule placement.

Subsequently, for both general positions and special positions, a series of structure checks is performed to ensure that no two molecules are unphysically close to each other.^86–88^ The specific radius, *s*_*r*_, is used as a measure of the distance between atoms of different molecules. The *s*_*r*_ is a fraction of the sum of the van der Waals radii, *r*_*A*_ and *r*_*B*_, of two atoms, *A* and *B*, belonging to different molecules. The distance, *d*_*A*,*B*_, must be such that *d*_*A*,_ *_B_* ≥*s*_*r*_(*r_A_* + *r*_*B*_). Otherwise, the structure is rejected. In Genarris, *s*_*r*_ is a user-defined parameter with a default value of 0.85. Smaller default *s*_*r*_ values have been assigned to strong hydrogen bonds, characterized by considerably shorter intermolecular distances than typical van der Waals interactions.^87^ Finally, valid structures are passed to Ogre to generate film slabs. If no surface cleavage is needed, this procedure is equivalent to standardizing the film unit cells by making the ***c*** lattice vector normal to the ***a***–***b*** plane, and wrapping the atoms inside the unit cell.

### 2.4 Down-Selection

Once a “raw” pool of film slab models is generated, the user may choose to reduce the number of structures to be considered further to avoid performing a large number of expensive DFT calculations. Genarris offers a down-selection procedure that relies on clustering by structural similarity to help form a smaller curated pool of structures without loss of diversity.^86–88^ The down-selection workflow implemented in Genarris Interfaces is shown in Figure 4.

**Figure 4:**
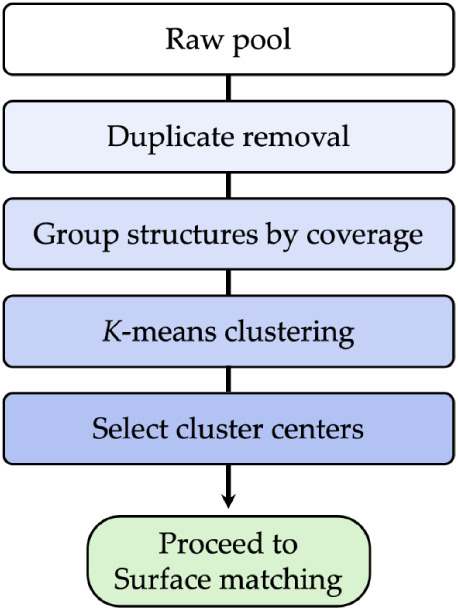
Workflow of clustering and down-selection of film structures.

Owing to the comparatively small number of layer groups and the constraints imposed by demanding commensurism with the substrate, many of the generated film structures are identical. Duplicate structures are removed with the StructureMatcher module of Pymatgen^101^ which performs unit cell standardization with Niggli reduction to eliminate equivalent unit cells. The fractional length tolerance, site tolerance, and angle tolerance are set to 0.1, 0.2, and 3 respectively, based on the empirical observation that with these settings duplicate structures are effectively removed with negligible loss of unique structures. This is a stricter tolerance than the default settings used in Genarris for molecular crystals because even interfaces with small structural differences can have significantly different energy and properties. The remaining structures are then grouped by coverage, calculated by dividing the number of molecules per cell by the area of the corresponding substrate supercell.

For each structure, an atom-centered symmetry functions (ACSF)^102,103^ representation is constructed with Dscribe,^103^ using the default settings recommended by the developers. The ACSF representation captures the local environment of each atom by using a fingerprint comprised of two-body and three-body functions. The ACSF descriptors of all atoms are concatenated to construct the representation of the unit cell structure. *K*-means clustering^104^ is then performed for each coverage. *K*-means clustering has been chosen because of its ease of implementation and computational efficiency. We have empirically observed that the structures belonging to the same cluster are typically very similar. Therefore, only the cluster centers are selected for the next step. The number of clusters (*k*) is defined by the user. We recommend 5–10 clusters per coverage for a comprehensive sampling of representative structures. After duplicate removal, clustering, and down-selection, the remaining structures proceed to the interface construction step.

### 2.5 Surface Matching

After clustering and down-selection the remaining film structures are paired with commensurate substrate structures to produce an interface. The substrate slabs are constructed using ASE with a user-defined thickness. The recommended best practice for determining the appropriate thickness is to evaluate the relative energies of representative interface structures as a function of the number of substrate layers (see examples in the SI). For all systems studied here, the substrate thickness is well converged at 3 layers, and the ranking does not change with the addition of more layers.

Genarris Interfaces does not assume a particular binding site of the molecules to the substrate surface or a particular orientation of the film molecules with respect to the substrate surface (film structures are generated with random orientations, as described above). Therefore, surface matching is performed to determine the optimal registry in the plane of the interface and the optimal distance between substrate and film in the direction perpendicular to the interface. This is performed for each pair of film and commensurate substrate. During surface matching the film is shifted with respect to the substrate, but its structure is kept rigid. The goal of the surface matching step is to produce a reasonable starting point for DFT relaxation.

The surface matching module in Genarris Interfaces is based on a similar module implemented in Ogre for inorganic interfaces.^98^ Therein, a geometric score function was formulated based on the overlap and empty space between atomic spheres at the interface. The geometric score function is very fast to evaluate. Here, we formulate a score function tailored for organic/inorganic interfaces in the same vein. The geometric score function is based on the scaled overlap volumes of attractive, *A̅*, and repulsive, *R̅*, regions at the interface:

2 $$S=c\times {\left(1+\mathrm{R̅}\right)}^{2}-\mathrm{A̅}$$

where the scaled attractive volume is defined as the overlap volume of the attractive regions of atomic spheres divided by the total volume occupied by atoms at the interface:

3 $$\mathrm{A̅}=\frac{V_{a}}{\underset{atoms}{\sum }V_{at}}{,r}_{a}=r_{vdw}+\alpha$$

The spheres that represent the attractive region are produced by the attractive radii, *r*_*a*_, obtained by adding a certain value, α, to the atomic van der Waals radii to approximate long-ranged attractive interactions. Similarly, the scaled repulsive volume is defined as the overlap volume of the repulsive regions of atomic spheres divided by the total volume occupied by atoms at the interface:

4 $$\mathrm{R̅}=\frac{V_{r}}{\underset{atoms}{\sum }V_{at}}{,r}_{r}=r_{vdw}-\beta$$

The spheres that represent the repulsive region are generated by the repulsive radii, *r*_*r*_, obtained by subtracting a certain value, β, from the atomic van der Waals radii to mimic short-ranged repulsive interactions. Here, we used empirically determined values of 0.15 for α and 0.25 for β. The value of *c* controls the overall shape of the score function, and is set as 4 here. These values can be modified by the user in the input configuration file. We recommend testing and adapting the parameters of the score function for each system.

The geometric score function serves as a surrogate model, which is faster to evaluate than the energy. Configuration space exploration is performed by using Bayesian optimization (BO) to maximize the objective function, defined as the negative of the score function. The film is shifted with respect to the substrate in the *x*, *y*, and *z* directions. The parameter bounds for shift values were set to the surface lattice parameters in the *XY* plane and 0–4 Å for the interfacial distance in the *Z* direction. To perform BO, we utilize the BayesianOptimization^105^ package. The BayesianOptimization library exploits Gaussian processes as surrogate models. For handling the exploration-exploitation trade-off, the upper confidence bound (UCB) acquisition function was utilized with a kappa value of 8, chosen to account for the vast size of the search space. We note that in more recent versions of Ogre the geometric score function has been replaced by a classical force field for inorganic interfaces between ionic materials^106^ and by a machine learned potential for organic interfaces.^85^ In the future, we also plan to explore such alternatives for the geometric score function in Genarris Interfaces.

### 2.6 Relaxation and Ranking

In the last step, the surface-matched interface structures are relaxed with FHI-aims as detailed in Section 3.1. We note that the conformation of adsorbed molecules can only change to the extent possible by local optimization. For molecules with rotatable bonds, film structure generation would have to be performed using different conformers. As discussed below, we find that in some cases the interface structure changes very significantly during DFT relaxation, owing to the interaction with the substrate. The relaxed interface structures form the final pool output by Genarris Interfaces. To compare the stability of structures with different coverage values, which have a different number of substrate atoms, the ranking is performed based on the adhesive energy defined as^106–109^

5 $$W_{ad}=\frac{\left(E_{substrate}+E_{film}-E_{interface}\right)}{A}$$

Where *E*_*substrate*_, *E*_*film*_ and *E*_*interface*_ refer to the DFT total energy of the isolated substrate, the isolated film, and the combined interface, respectively. *A* is the surface area of the interface. Adhesive energies were computed based on total energies calculated with PBE+TS^surf^, and higher adhesive energies indicate more stable interface.

## 3 Computational Details

### 3.1 DFT Settings

Genarris Interfaces is compatible with the FHI-aims^92^ electronic structure code for energy evaluation and geometry relaxation of interface structures. Relaxations were performed using the generalized gradient approximation of Perdew–Burke–Ernzerhof (PBE).^110^ Dispersion interactions were treated with the Tkatchenko–Scheffler (TS)^111^ pairwise dispersion correction, modified for describing the interaction of adsorbates on surfaces by incorporating the Lifshitz–Zaremba–Kohn (LZK) theory for describing the screening of the substrate’s electrons (TS^surf^).^112^ DFT+TS^surf^ has been shown to provide accurate results for the binding distance and binding energy of organic molecules on coinage metals.^112,113^

The *light* numerical setting and *tier 1* basis sets of FHI-aims^92^ were used for all calculations. A convergence test comparing to *tight*/*tier 2* settings is provided in the SI. To converge the *k*-point grid for single point energy (SPE) evaluations, the number of *k*-points in the plane of the interface was increased until no further changes were obtained in the relative energy ranking among structures. A full account of these convergence tests is provided in the SI. Based on this, a 6 × 6 × 1 *k*-point grid was used for SPE calculations and a 4 × 4 × 1 *k*-point grid was used for geometry relaxation. All geometry relaxations were conducted with the Broyden–Fletcher–Goldfarb–Shanno (BFGS)^114^ optimization algorithm until the remaining forces were below 0.01 eV/Å, which is expected to yield an uncertainty of about 0.06 Å in the adsorption geometry.^73^ Lindh Hessian initialization^115^ was applied in geometry optimization to improve convergence.^73^ The lattice parameters were fixed during geometry optimization. All the film atoms and the first layer of substrate atoms were free to move. The Heyd, Scuseria, and Ernzerhof (HSE)^116^ hybrid functional was utilized for subsequent electronic structure calculations of selected interfaces with a *k*-point grid of 4 × 4 × 1.

### 3.2 STM Simulations

Scanning tunneling microscopy (STM) is an experimental technique that probes the electron density at the surface of a material by applying a bias to the sample, forcing electrons to tunnel from the surface to the STM tip or vice versa depending on the sign of the bias.^117^ Although STM does not directly probe the surface structure, it has been shown that the surface structure is directly linked to the electron density.^118^ Therefore, DFT can be used to simulate STM images and compare with experiment to gain a deeper understanding of the exact structure of a surface. We have developed CubeSTM to simulate STM images from partial charge files. CubeSTM utilizes a modified Tersoff–Hamann^117^ approach, considering three additional effects: nonlocal tunneling, screening, and electron localization, to create images that closely resemble experimental images. A detailed description of the CubeSTM package is provided in the SI. The electron densities of all eigenstates between the Fermi level and the bias applied in the experiment are summed up and output in a CUBE file format, which serves as the input for CubeSTM. Here, this was performed using FHI-aims^92^ with a 4 × 4 × 1 *k*-point grid. The applied voltage used in our simulations was 0.1 V, −0.34 V, and −2.0 V for TCNE/Au(111), PTCDA/Ag(111) and Naphthalene/Cu(111) respectively, in accordance with the experimental setups.^119–121^

## 4 Results and Discussion

### 4.1 PTCDA on Ag(111)

Perylenetetracarboxylic dianhydride (PTCDA) is a π-conjugated organic semiconductor that has been extensively studied as a representative system for understanding organic thin films.^122,123^ Several studies have investigated its adsorption sites and changes to the molecular conformation upon adsorption.^44,99,124–128^ When deposited on various close-packed substrates, including Ag(111), PTCDA typically forms a flat-lying commensurate monolayer with two molecules occupying inequivalent adsorption sites in a herringbone pattern that closely resembles the (102) plane of the bulk β crystal form^122,123,129^ Owing to the low-lying *d*-band of Ag and the high mobility of PTCDA molecules at a sufficiently high temperature,^45^ PTCDA molecules are able to maintain contact with the Ag(111) surface and diffuse over large distances to form an ordered, commensurate film on the Ag substrate.

Figure 5 shows the distributions of the γ-angle and interface area for PTCDA film structures generated by Genarris Interfaces in successive down-selection stages. The parameters of the experimentally observed structure, with a γ-angle of 90° and an interfacial area of 2.39 nm^2^,^120^ are indicated by the intersection of the two red dashed lines. The raw pool, shown in panel a, contains 4626 film structures with γ-angles ranging from 30.2° to 149.9° and interface areas ranging from 0.37 nm^2^ to 4.87 nm^2^. Most of the generated structures have a γ angle of 90° because most compatible layer groups for *Z* = 2 are rectangular. Panel a reveals a discrete distribution of the interface areas, where the interface area of the most frequent bin is about twice the area of less frequent bins. This indicates an integer-multiple relationship of the corresponding epitaxy matrices. The larger interface area bin is more populated because molecules are more likely to overlap in smaller unit cells, making the structure generation more difficult. After duplicate removal (panel b), 1,571 unique structures remain, most of which are still concentrated in the most frequently sampled area bins. After *k*-means clustering and selection of representative structures, the γ-angle and interface area distributions become more uniform, due to the removal of similar structures, as shown in panel c. After this step, 245 structures remain in the pool, which proceed to surface matching and DFT relaxation.

**Figure 5:**
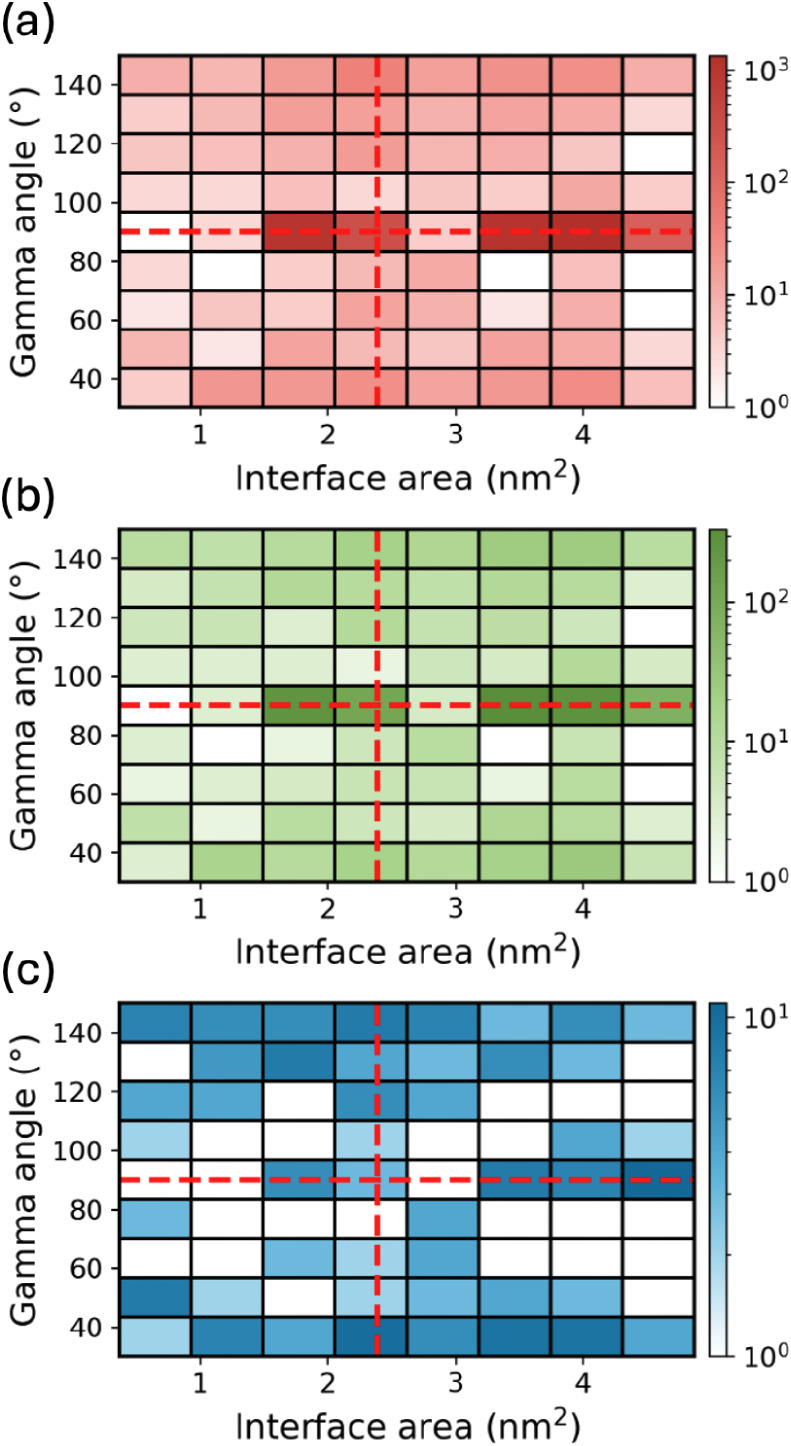
Joint distribution of the γ-angle and interface area of PTCDA film structures after key steps of the structure generation and down-selection process: (a) film slab construction, (b) duplicate removal, and (c) *k*-means clustering. The experimental γ-angle (90°) and interface area (2.39 nm^2^) are indicated by red dashed lines.

In Figure 6a the adhesive energies of the final relaxed PTCDA/Ag(111) interface structures are plotted as a function of coverage. At low coverage it is energetically favorable for PTCDA to form a monolayer of molecules that lie flat on the Ag(111) surface. A previous experimental study has shown that a flat-lying adsorption mode results in the greatest charge transfer rate for a single PTCDA molecule on an Ag(111) substrate.^130^ Such “planar monolayer” structures, colored in blue in Figure 6a, form an approximately linear front. Among the structures with the coverage closest to that of the experimental structure, the one with the greatest adhesive energy, circled in orange and shown in panel b, is a planar monolayer structure that resembles the experimental structure. This structure is stabilized by weak intermolecular CH···O hydrogen bonds in addition to molecule–surface interactions.

**Figure 6:**
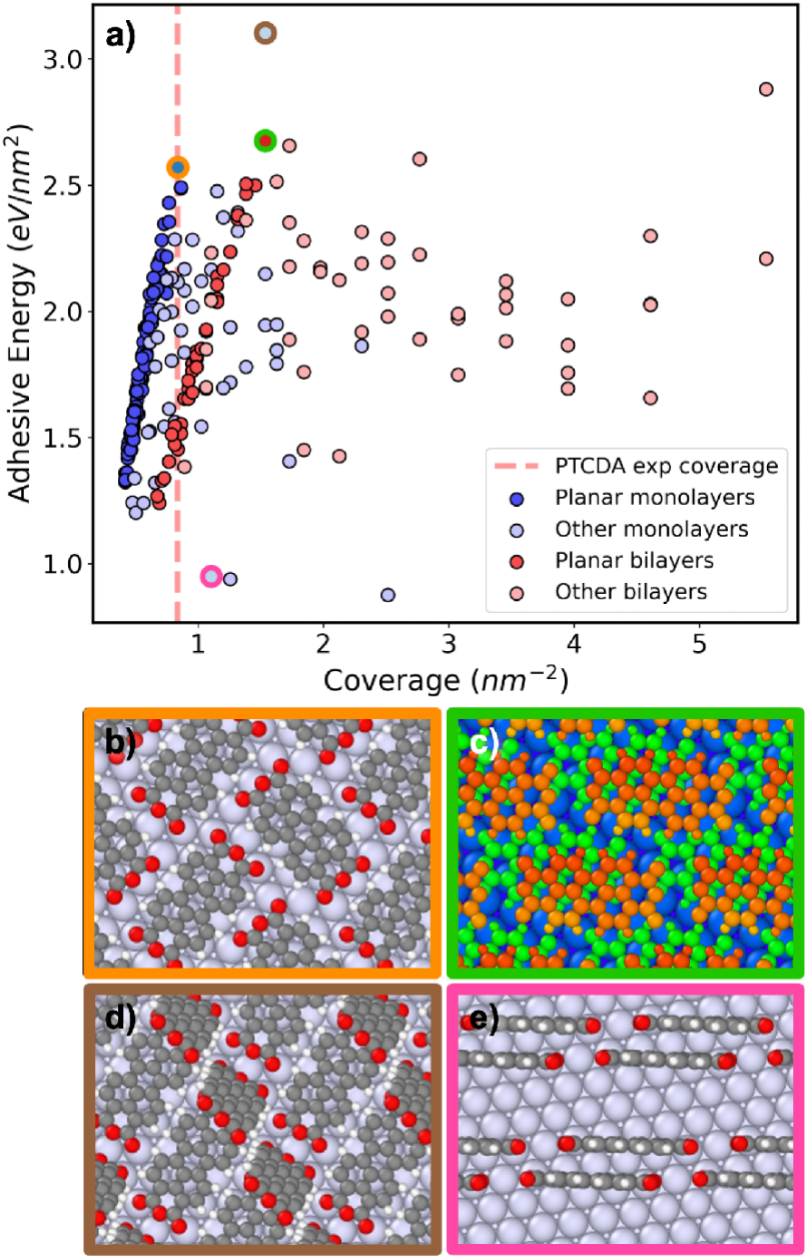
(a) Adhesive energy as a function of coverage for the final pool of relaxed PTCDA/Ag(111) structures. Structures are colored according to the number of molecular layers and whether all molecules lie flat on the surface. (b) The structure with the highest adhesive energy at the experimental coverage of approximately 0.837 nm^–2^ (circled in orange in panel a). (c) The most stable planar bilayer structure (circled in green in panel a). (d) The structure with the highest adhesive energy overall (circled in brown in panel a). (e) An unfavorable structure with the molecules adsorbed on the long edge (circled in pink in panel a).

At higher coverage, bilayer structures composed of two layers of flat-lying molecules become more favorable. These planar bilayer structures, colored in red in Figure 6a, also form an approximately linear front. The most stable structure of this type is circled in green and shown in panel c with the molecules belonging to the top and bottom layers colored in red and green, respectively. As the coverage increases, structures with nonplanar adsorption modes become more frequent. Monolayer structures with nonplanar adsorption modes, colored in light blue in Figure 6a are found mainly in the region between the planar monolayer and bilayer fronts, and slightly past the bilayer front. Bilayer structures with nonplanar adsorption modes, colored in light red, become more frequent as the coverage increases past ∼ 2 nm^–2^. The structure with the greatest adhesive energy overall, circled in brown and shown in panel d, has a coverage of 1.537 nm^–2^. This structure consists of one molecule lying flat on the substrate and a second molecule that is nearly perpendicular to the surface, adsorbed only via the anhydride group. Despite the unreasonable appearance of this structure, its adhesive energy is persistently the highest using different DFT functionals and dispersion methods, as shown in the SI. Genarris Interfaces also generates some structures with other adsorption modes. For example, the structure circled in pink and shown in panel e, which is energetically unfavorable, comprises upright molecules adsorbed via their longer edge.

Figure 7 shows a comparison between the experimental STM image of PTCDA/Ag(111)^120^ and the simulated STM image of the most stable structure near the experimental coverage, circled in orange in Figure 6a and illustrated in Figure 6b. A complete account of the CubeSTM settings used to generate this figure is given in the SI. The structure generated by Genarris Interfaces is in close agreement with the experimentally observed structure in terms of the lattice vectors and the molecular arrangement in the unit cell.

**Figure 7:**
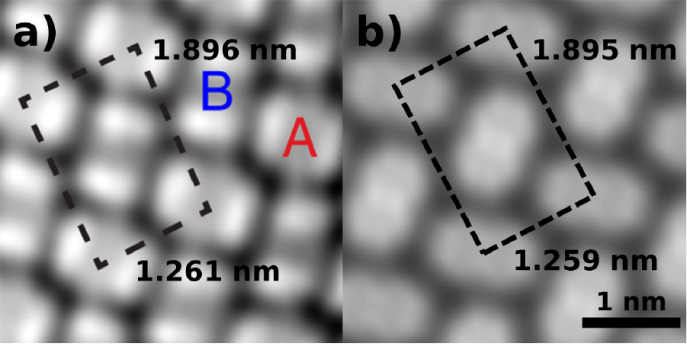
Comparison between the experimental structure of PTCDA/Ag(111) and a structure generated by Genarris Interfaces. (a) An STM image of an experimental PTCDA/Ag(111) structure taken at *V_b_* = −340 mV, reproduced with permission from ref 120 Copyright 2018 American Physical Society. (b) A simulated STM image of the PTCDA/Ag(111) structure shown in Figure 6b. Gaussian blurring is applied to simulate the resolution of the experiment.

The structure of the interface affects its electronic properties. In Figure 8, the computed density of states (DOS) for the interface structure generated by Genarris Interfaces is compared to an ultraviolet photoemission (UPS) experiment performed on a 2 Å thick PTCDA film on Ag(111).^131^ DFT simulations of PTCDA and similar compounds have been shown to be particularly sensitive to the choice of the exchange-correlation functional due to the self-interaction error (SIE), which arises from the spurious repulsion of an electron from its own charge density. With semi-local functionals, such as PBE, the orbitals localized on the anhydride groups are destabilized and shifted to higher energies compared to orbitals delocalized over the perylene core. This issue can be mitigated by the inclusion of Fock exchange in hybrid functionals.^132–135^ For this reason, we used the HSE hybrid functional to study the electronic structure of the PTCDA/Ag(111) interface.

**Figure 8:**
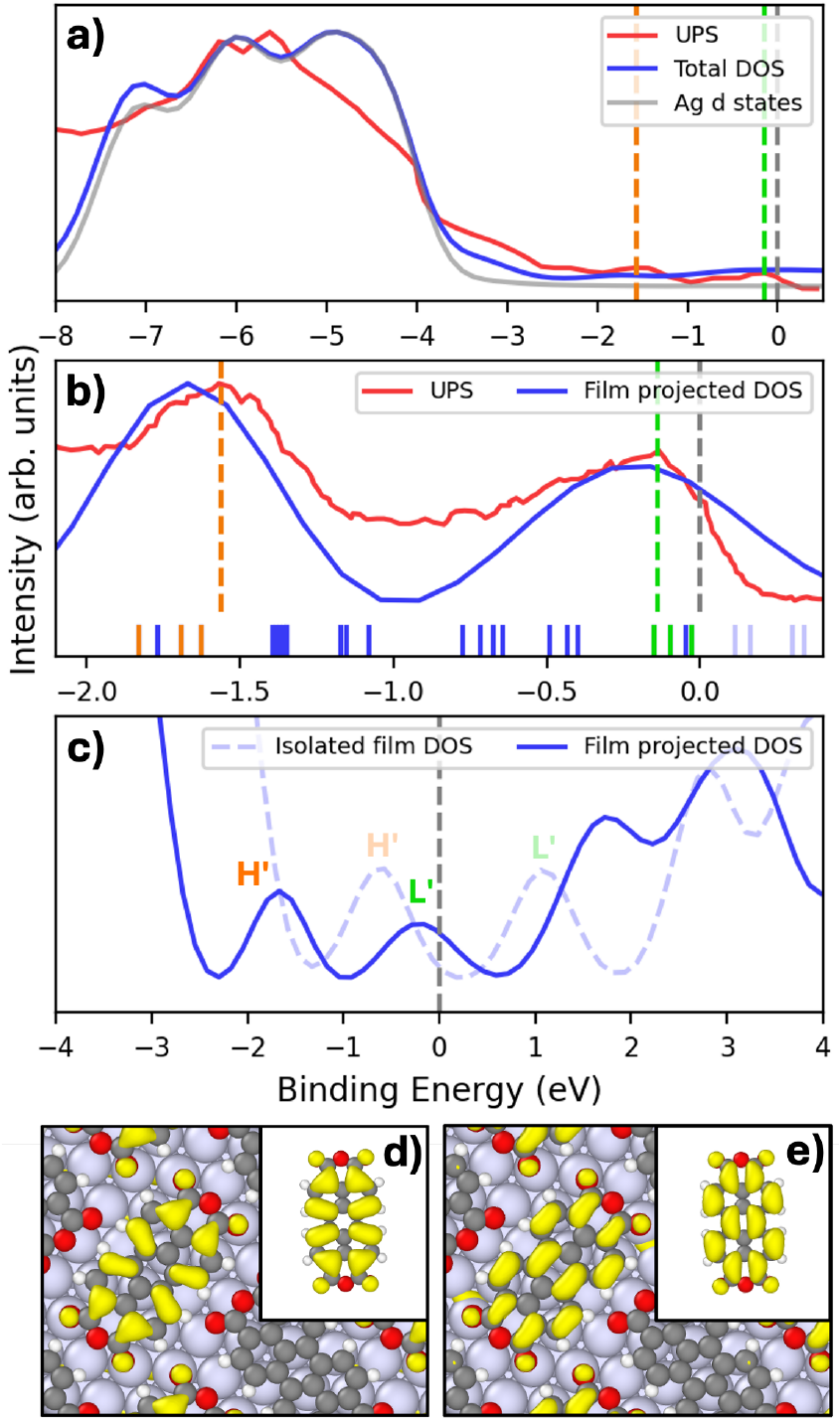
Electronic structure of PTCDA/Ag(111): (a) HSE DOS of the best matched interface structure generated by Genarris, compared with UPS data adapted with permission from ref 131 Copyright 2008 Elsevier. A broadening of 0.3 eV is applied to all DFT DOS curves to simulate the resolution of the experiment. The Fermi level is indicated by the gray dashed line. The HOMO-derived peak (H′) and LUMO-derived peak (L′) are indicated by the orange and green dashed lines, respectively. (b) A magnified view of the HOMO–LUMO region, also showing the corresponding discrete eigenstates, indicated by tick marks. The H′ and L′ states are highlighted in orange and green, respectively. All other occupied states are colored in blue and unoccupied states above the Fermi level are colored in light blue. (c) Comparison of the DOS of the isolated PTCDA film and the film projected DOS of the interface. (d) A HOMO-derived state of the interface. The inset shows the HOMO of an isolated molecule. (e) A LUMO-derived state of the interface. The inset shows the LUMO of an isolated molecule.

Panels a and b in Figure 8 show the HSE DOS compared to UPS.^131^ The peak assigned to the HOMO-derived states (H′) and the peak assigned to the LUMO-derived states (L′) in ref 131 are indicated by orange and green dashed lines, respectively. A decomposition of the interface DOS shows that the Ag-*d* states contribute predominantly to the large peak observed between −8 eV to −3.5 eV, obscuring signals from the PTCDA film in that region. A comparison of the film-projected DOS to UPS in the HOMO–LUMO region is shown in panel b. Isosurfaces of eigenstates of interest were visualized to identify the PTCDA H′ and L′ states. As shown in Figure 8, the interface H′ and L′ states resemble the respective molecular states. The assignment of the peaks observed in the UPS is confirmed by the good agreement with the film-projected DOS and the corresponding H′ and L′ states, indicated by orange and green tick marks, respectively. Owing to charge transfer from the Ag substrate, all L′ states, the highest of which is located near the Fermi level, are occupied. This makes the PTCDA/Ag interface metallic, in agreement with experiment. A comparison between the isolated PTCDA film DOS and the film projected DOS of the interface, shown in panel c, further elucidates the effect of charge transfer from the Ag substrate. For the isolated PTCDA film, the H′ states are found below the Fermi level and are occupied, whereas the L′ states are found above the Fermi level and remain unoccupied. As a result of charge transfer from the Ag substrate, the L′ states become occupied and the Fermi level is shifted. Thus, the electronic structure of the best matched PTCDA/Ag interface structure generated by Genarris is in close agreement with a UPS experiment. The computed DOS helps reveal the effect of charge transfer and assign the observed peaks to specific interface states.

### 4.2 TCNE on Au(111)

Tetracyanoethylene (TCNE) on metal surfaces is a prototypical organic/inorganic interface used to study charge transfer interactions at the molecular scale. As a strong electron acceptor, TCNE exhibits substrate-dependent electron transfer and hybridization with the metal states, yielding strong interface dipoles and substantial modification of the work function.^74,136,137^ Depending on the surface and growth conditions, TCNE can adopt distinct adsorption geometries and form surface-induced polymorphs that differ in their molecular packing, electronic properties, and interfacial energetics.^119,138^ On more reactive surfaces, such as Cu(100), surface reconstruction can be induced by adsorption.^139^ An STM study of a single TCNE molecule on Cu(111) has demonstrated tip-induced switching between five states, one of which is magnetic.^140^ This makes TCNE/metal systems interesting for studying the relationship between the interface structure and its electronic properties.

Figure 9 shows the interface area distribution of TCNE film structures generated by Genarris Interfaces after key steps of the structure generation and down-selection process. With three molecules per film unit cell, all allowed layer groups have hexagonal symmetry. Therefore, all the generated structures have γ = 120°, which agrees with experiment.^119^ The area distribution is discrete due to the imposition of commensurability. The raw pool of film slab structures (panel a) consists of 5200 structures with interface areas ranging from 0.65 nm^2^ to 2.66 nm^2^. A significant fraction of the generated film structures are close to the experimental area, which is indicated by the red dashed line at 2.24 nm^2^.^119^ In general, it is easier to generate structures with larger area and lower coverage, which manifests in the lower frequency of structures with smaller areas. After duplicate removal (panel b), 214 structures remain in the pool, 52 (24%) of which are in the experimental area bin. The area distribution remains similar to that of the raw pool. After *k*-means clustering (panel c), 82 film structures remain, 9 (11%) of which are in the experimental area bin, and the area distribution becomes more uniform. These structures proceed to surface matching and DFT relaxation.

**Figure 9:**
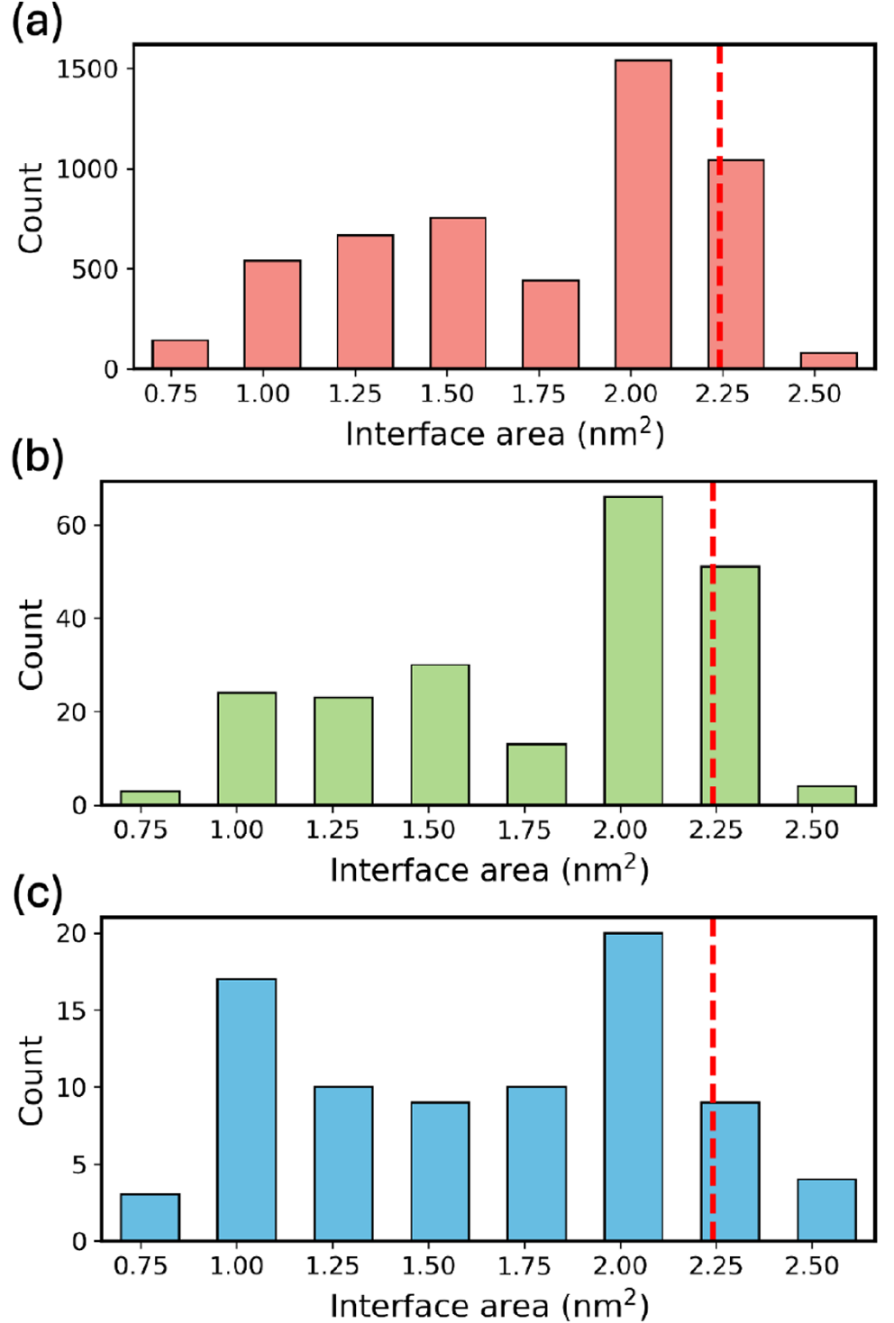
Interface area histograms of TCNE film structures after key steps of the structure generation and down-selection process: (a) film slab construction, (b) duplicate removal, and (c) *k*-means clustering. The experimental interface area of 2.24 nm^2^ is indicated by the red dashed lines.

Figure 10a shows the adhesive energy as a function of coverage for the final relaxed interface structures. The adsorption configurations of the TCNE molecules can be categorized into “planar” (molecules lie flat), “upright” (molecules lie on their edges, orthogonal to the surface), and “inclined” (all other tilted configurations). The upright adsorption configurations can be further subdivided according to whether the C=C bond in TCNE is parallel or perpendicular to the surface. Planar structures are only found at low coverage, below 1.667 nm^–2^, and are energetically favorable throughout that range. At higher coverage, crowding makes planar configurations less likely, which has been observed experimentally in TCNE/Cu(111).^141^ Representative planar structures with the highest adhesive energies at the experimental coverage of 1.338 nm^–2^ are circled in orange and brown and shown in panels b and c. Upright structures are energetically unfavorable across most of the coverage range studied here. A representative upright structure with the experimental coverage is circled in cyan and shown in panel d. Two additional upright structures with perpendicular and parallel adsorption modes and a coverage of 3.204 nm^–2^ are circled in purple and pink, respectively, and shown in panels e and f. Only at very high coverage the upright adsorption mode becomes energetically favorable. The upright structure, circled in green and shown in panel g, has the highest coverage and the highest adhesive energy overall.

**Figure 10:**
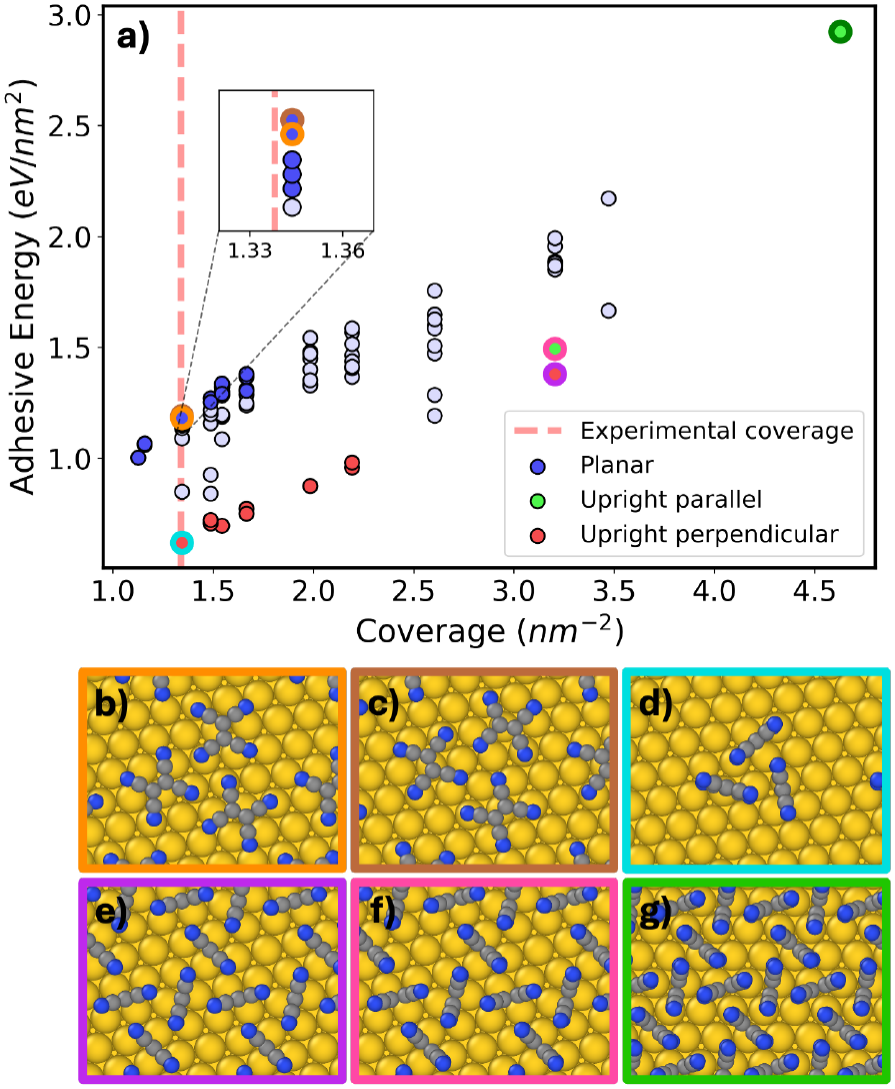
(a) Adhesive energy as a function of coverage for the final pool of relaxed TCNE/Au(111) structures. Structures are colored according to molecular orientation with respect to the substrate. (b, c) The structures with the highest adhesive energies at the experimental coverage of 1.338 nm^–2^ and a planar adsorption mode (circled in orange and brown in panel a). (d) A structure with the experimental coverage and an upright perpendicular adsorption mode (circled in cyan in panel a). (e, f) Analogous structures with upright perpendicular and upright parallel adsorption modes and a coverage of 3.204 nm^–2^ (circled in purple and pink, respectively, in panel a). (g) The structure with the highest coverage and highest adhesive energy overall (circled in green in panel a).

Figure 11 shows a comparison between simulated STM images of structures generated by Genarris Interfaces and the experimentally observed structure.^82^ A full account of the CubeSTM settings used to produce the simulated images is provided in the SI. Panels b and c show the planar structures, circled in orange and brown in Figure 10a and visualized in Figure 10b,c, respectively. Panel d shows the upright perpendicular structure with the experimental coverage, circled in cyan in Figure 10a and visualized in Figure 10d. These three structures have very close unit cell dimensions to the experimental structure and possess a similar triangular spiral packing motif. The triangular spirals of the planar structures come closest to each other at their vertices, producing dark triangular gaps that resemble those in the experimental STM image. The triangular spirals of the upright perpendicular structure in panel d are farther apart than in the experimental image. In refs 82, 119 the adsorption mode of TCNE on Au(111) was assigned as upright parallel based on the shape of the STM blobs. For comparison of the blob shapes produced by different adsorption modes, panels e and f show the upright perpendicular and upright parallel structures obtained at a higher coverage (3.204 nm^–2^), which are circled in purple and pink in Figure 10a and visualized in Figure 10e,f, respectively. These structures have significantly smaller unit cells than the experimental structure and the dark gaps between molecules are hexagonal rather than triangular. The blobs formed by the upright perpendicular molecules are somewhat more elongated, whereas the blobs formed by the upright parallel molecules are more rounded. The blobs formed by the planar molecules in panels b,c are perhaps somewhat more rectangular, but distinguishing between the adsorption modes based on the blob shape may be difficult.

**Figure 11:**
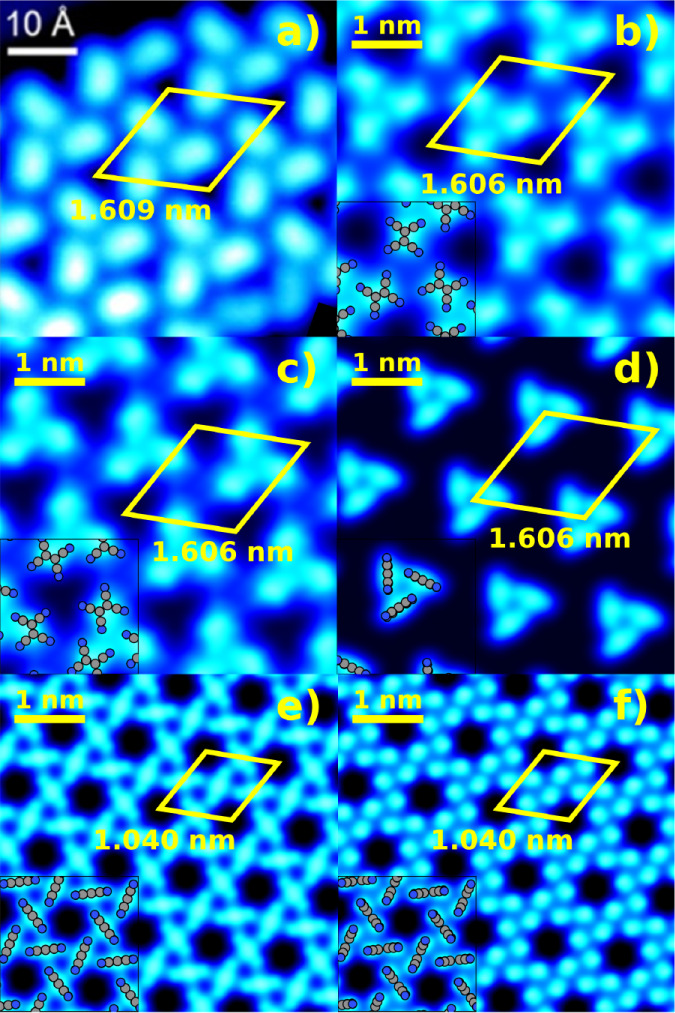
Comparison between simulated STM images of structures generated by Genarris Interfaces and the experimentally observed structure of the TCNE/Au(111) interface: (a) STM image reproduced with permission from ref 82 Copyright 2017 American Chemical Society; (b) the structure that most resembles the experimental STM, but with a planar adsorption mode, shown in Figure 10b; (c) the structure with the highest adhesive energy at the experimental coverage, shown in Figure 10c; (d) the structure with the experimental coverage and upright-perpendicular molecules, shown in Figure 10d; and two analogous structures with higher coverage and (e) upright perpendicular and (f) upright parallel adsorption modes, shown in Figure 10e,f, respectively.

An earlier computational study employed the SAMPLE code to generate TCNE/Au(111) interface structures.^82^ Therein, the experimental structure was used as a starting point. Hence, structure generation was constrained to the experimental coverage and the film packing motif was further constrained to triangular configurations. Within the SAMPLE approach the adsorption mode is inherently constrained because interface unit cells are constructed by combining subunits with one molecule adsorbed on the surface (this is the central difference between SAMPLE and Genarris). The choice of exchange-correlation functional and dispersion method can affect the relative stability of different adsorption configurations.^113^ In ref 82 stability ranking was based on total energy (rather than adhesion energy) calculated using PBE with the many-body dispersion (MBD)^142,143^ correction. A final difference between ref 82 and the present study is that they used a less stringent force convergence threshold of 0.1 eV/Å for relaxation.

With these differences in mind, in ref 82 a similar structure to the one shown in [Fig fig11]b, but with an upright parallel adsorption mode, was ranked as the most stable. The SI of ref 82 also shows that structures with a planar adsorption mode, similar to the ones shown in [Fig fig11]b,c, were ranked among the most stable. In our final pool of interface structures, no interface with the experimental coverage has an upright parallel configuration. With the force convergence threshold used here, the vast majority of structures initially generated with an upright parallel configuration changed their adsorption mode upon relaxation. In the SI, we provide examples demonstrating that structures generated with an upright parallel adsorption mode relax to a planar configuration with our relaxation settings, but remain upright with the settings used in ref 82. We additionally provide in the SI an analysis of the effect of the DFT method on the stability ranking, showing that the planar structures are persistently ranked as the most stable (with the highest adhesion energy).

Despite the differences between the structure generation approach and the method used for relaxation and ranking here and in ref 82, both studies agree on the fact that an exact match to the experimentally observed structure of TCNE/Au(111) is not found among the most stable structures. This discrepancy could be attributed to the limitations of the DFT methodology used in both studies or to thermal and kinetic effects, not considered in either study. In ref 82 a structure closely resembling the experimentally observed packing was produced only when an Au adatom was placed at the center of each triangle of TCNE molecules. Hence, this does not appear to be a limitation of Genarris Interfaces.

We further investigate the dependence of the electronic structure of the TCNE/Au(111) interface on the adsorption mode and coverage. In Figure 12 the computed HSE DOS of different interface structures are compared to the scanning-tunneling spectroscopy (STS) experiment from ref 119 shown in panel (a) with the peak assignment suggested therein. In order to differentiate the contributions from the film and the substrate, the interface DOS and the film-projected DOS of each structure are shown in panels (b)–(e), and the DOS of the isolated substrate, which is the same for all interface structures, is shown in panel (f). The states derived from the molecular HOMO and LUMO states are denoted as H′ and L′, respectively. These were identified by visual inspection, as shown in panels (g),(h). The position of the highest H′ state is indicated by the orange dashed lines and the position of the lowest L′ state is indicated by the green dashed lines. To compare the spectral shape, we shifted the experimental STS measurement, such that its L′ peak aligns with the L′ peak of the most stable structure with the experimental coverage. The occupation of the L′ states depends on the adsorption mode, as shown in the insets of panels (b)–(e). For planar adsorption, all the L′ states remain unoccupied, whereas for upright adsorption (either parallel or perpendicular), the L′ states are partially occupied. Changing the adsorption mode from planar to upright additionally manifests in differences in the spacing between the LUMO-derived states. These differences are present in the isolated film DOS, shown in the SI, therefore they may be attributed to intermolecular interactions, rather than the interaction with the substrate. The adsorption mode also affects the position of the highest HOMO-derived peak. For the planar adsorption mode, shown in panel (b), the highest HOMO-derived state aligns with the peak attributed to an Au surface state in ref 119. For the structures with the upright adsorption mode, the peak corresponding to the H′ states appears in between two pronounced peaks originating from the Au substrate, the first of which aligns with the peak attributed to an Au surface state in ref 119. In all three cases, the peak attributed to the HOMO in ref 119 is aligned with the second large Au-derived DOS peak. Overall, the interface DOS varies only slightly with the adsorption mode and coverage, owing to the dominant contribution of the Au substrate. The differences in the spectral signatures of different interface structures may be too subtle to unambiguously assign a measured spectrum to a particular configuration.

**Figure 12:**
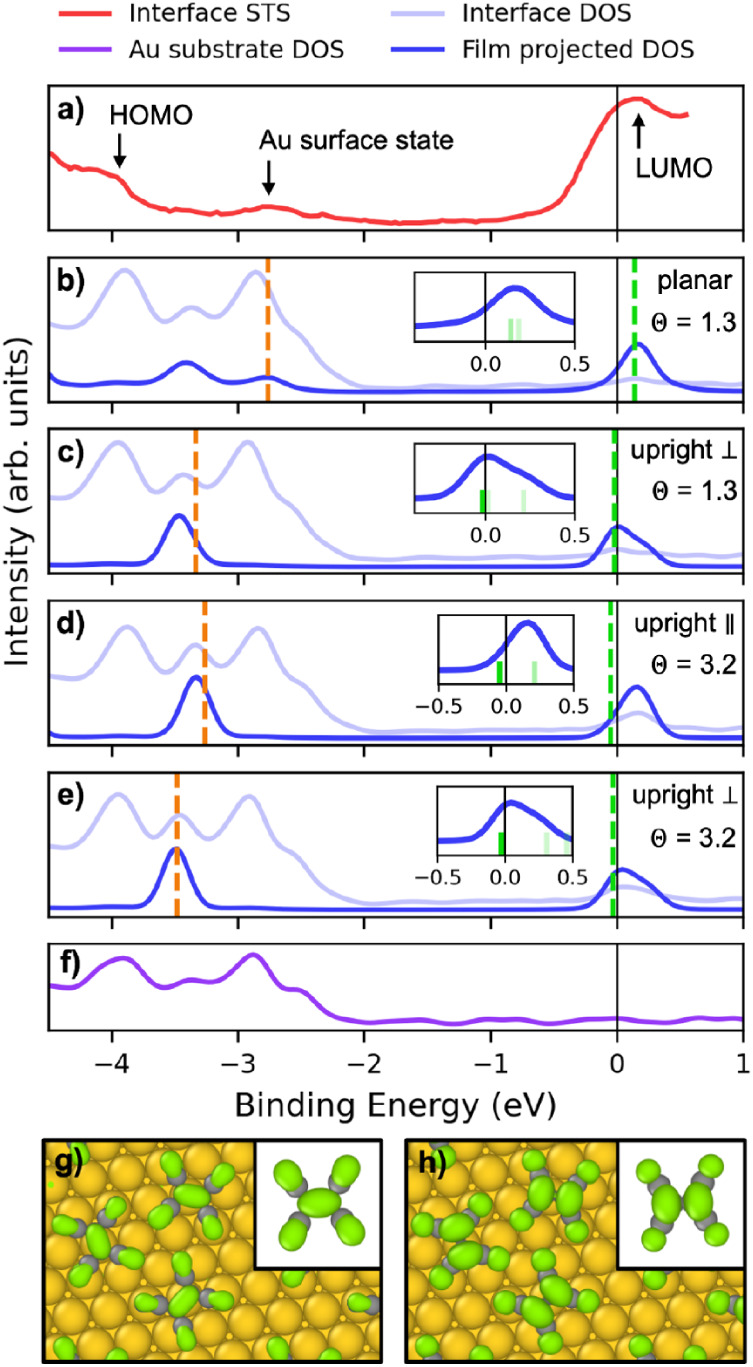
Electronic structure of the TCNE/Au(111) interface. (a) STS data reproduced with permission from ref 119 Copyright 2008 American Chemical Society. The peak assignment is taken from ref 119. Their assignment of the H′ state is inconsistent with the position of the H′ state in our simulations. (b)–(e) DOS of interface structures generated with Genarris and the corresponding film projected DOS: (b) the structure with the highest adhesive energy and planar molecules at the experimental coverage, shown in Figure 10c; (c) the structure with the lowest adhesive energy and upright-perpendicular molecules at the experimental coverage, shown in Figure 10d; and two structures with higher coverage and (d) upright-parallel and (e) upright-perpendicular adsorption modes, shown in Figure 10f,e, respectively. The highest H′ state and the lowest L′ state are indicated by orange and green dashed lines, and the experimental STS measurement is shifted such that its L′ peak is aligned with that of panel b. The insets in (b)–(e) show the discrete L′ states of the interface, and the occupied/unoccupied states are marked in green/light green, respectively. (f) DOS of the Au substrate. Visualizations of the interface (g) H′ and (h) L′ states. The insets show the corresponding single molecule HOMO and LUMO states.

### 4.3 Naphthalene on Cu(111)

Naphthalene is known to form ordered layers on various metal surfaces.^144–152^ On Cu(111), the formation of three ordered phases of naphthalene has been observed under different conditions of substrate temperature and adsorbate concentration.^121,153–155^ At a temperature of 120 K a phase with *Z* = 1, a γ-angle of 90°, and an interfacial area of 0.7 nm^2^ forms. A second phase with *Z* = 1, a γ-angle of 139°, and an interfacial area of 1.3 nm^2^ forms at a temperature of 140 K with a lower effective molecular concentration. A third phase with *Z* = 6, a γ-angle of 120°, and an interfacial area of 4.43 nm^2^ forms when the substrate is held at *T* = 120 K at a higher coverage.^121^ We note that we extracted the γ-angles and interfacial areas of the experimental structures directly from digitized STM images rather than using the idealized structures proposed in ref 121. The low-coverage structure observed at 140 K would not be generated with the default settings of Genarris because the machine learned model used for unit cell volume estimation was trained to predict the molecular solid form volume of close-packed crystal structures.^93^ For *Z* = 1, the model would predict a maximum interface area of 1 nm^2^, thus excluding the 140 K structure. Therefore, in order to generate structures with low coverage, we manually set the mean interface area to 1 nm^2^, with a deviation of 0.5 nm^2^.

Figure 13 shows the γ-angle and interface area distributions of naphthalene film structures with *Z* = 1 generated by Genarris Interfaces in successive stages of structure generation and down-selection. The bins corresponding to the experimental structures observed at 120 and 140 K are indicated by blue and red dashed lines, respectively. For *Z* = 1, all layer groups are compatible, enabling the generation of a wide range of γ-angles. The raw pool (panel a) contains 1536 structures. The γ-angle of 90° is the most frequently sampled because it is the most common value among all layer groups. In contrast, the γ-angle of 139° is less common. After duplicate removal (panel b) 551 structures remain in the pool. The angle distribution becomes somewhat more uniform, with a larger fraction of the structures having angles other than 90°, and the angle of 139° is well-represented. After *k*-means clustering (panel c), 248 structures remain in the pool. The fraction of structures with γ = 90° is further reduced and the area distribution becomes more uniform. The remaining structures proceed to surface matching and relaxation with DFT.

**Figure 13:**
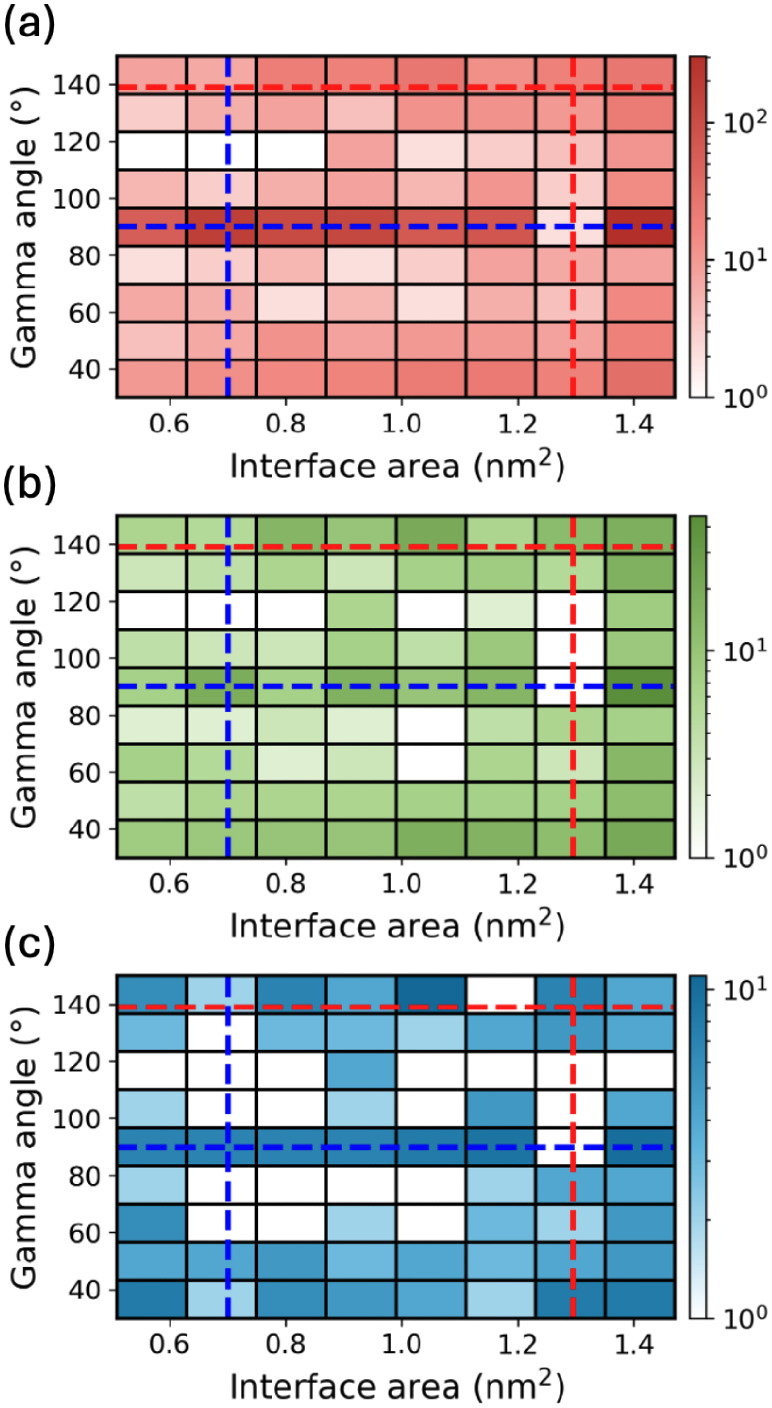
Joint distribution of the γ-angle and interface area of naphthalene film structures with *Z* = 1 after key steps of the structure generation and down-selection process: (a) film slab construction, (b) duplicate removal, and (c) *k*-means clustering. The structure observed at 120 K has a γ-angle of 90° and interface area of 0.7 nm^2^, indicated by the blue dashed lines. The structure observed at 140 K has a γ-angle of 139° and interface area of 1.3 nm^2^, indicated by the red dashed lines.

Figure 14a presents the adhesive energy as a function of coverage for the fully relaxed naphthalene/Cu(111) interface structures with *Z* = 1. Consistent with the trends observed for PTCDA/Ag(111) and TCNE/Au(111), planar structures with the molecules lying flat on the surface are favored at low coverage, as indicated by their higher adhesive energies. The upright adsorption mode is unfavorable throughout the coverage range generated here. The most stable structure overall, shown in panel b, has a coverage of 1.77 nm^–2^. This is the highest coverage for which planar monolayer structures are generated (with *Z* = 1 it is not possible to generate multilayer structures). The coverage values corresponding to the structures observed at 120 and 140 K are indicated by blue and red dashed lines, respectively. Distinct molecular arrangements are reported in ref 121 at 120 and 140 K.

**Figure 14:**
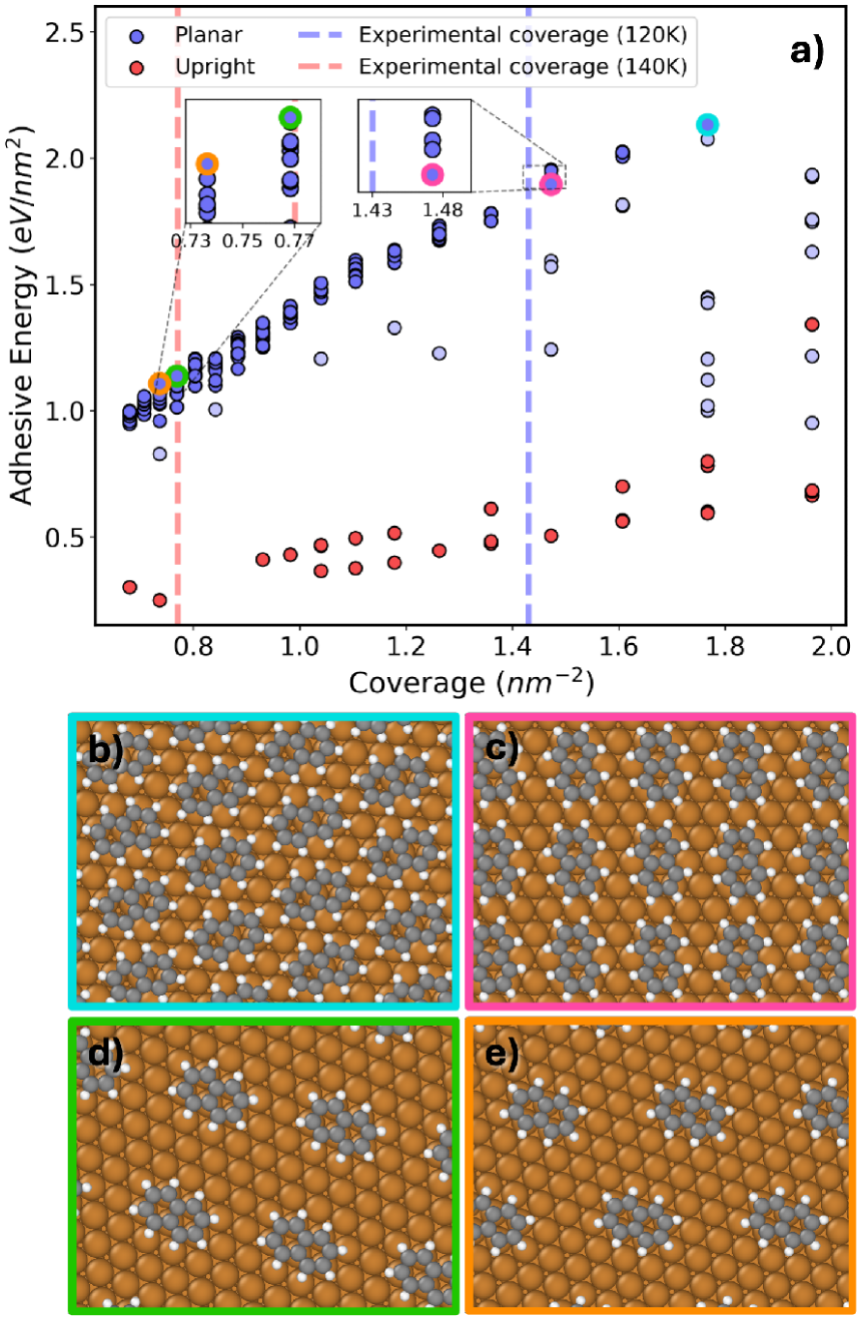
(a) Adhesive energy as a function of coverage for the final pool of relaxed naphthalene/Cu(111) structures with *Z* = 1. Structures with planar and upright adsorption modes are colored in blue and red, respectively. (b) The structure with the highest adhesive energy overall, circled in cyan in panel a. (c) The structure that most closely resembles the experimental structure at *T* = 120 K, circled in pink in panel a. (d) The most stable structure with the experimental coverage at *T* = 140 K, circled in green in panel a. (e) The structure that most closely resembles the experimental structure observed at *T* = 140 K, circled in orange in panel a.

The 120 K structure, whose STM image is shown in Figure 15a, exhibits a rectangular unit cell with a surface area of 0.7 nm^2^. In the coverage bin closest to this value, with a surface area of 0.69 nm^2^, there is a cluster of five planar structures with very close adhesion energies, ranked as the most stable. The four top structures in this cluster, shown in the SI, do not have a rectangular unit cell. The fifth structure in this cluster, highlighted in pink in Figure 14a and shown in panel c, has a rectangular unit cell that closely matches the observed structure. Its simulated STM image is presented in Figure 15b, showing excellent agreement with experiment.

**Figure 15:**
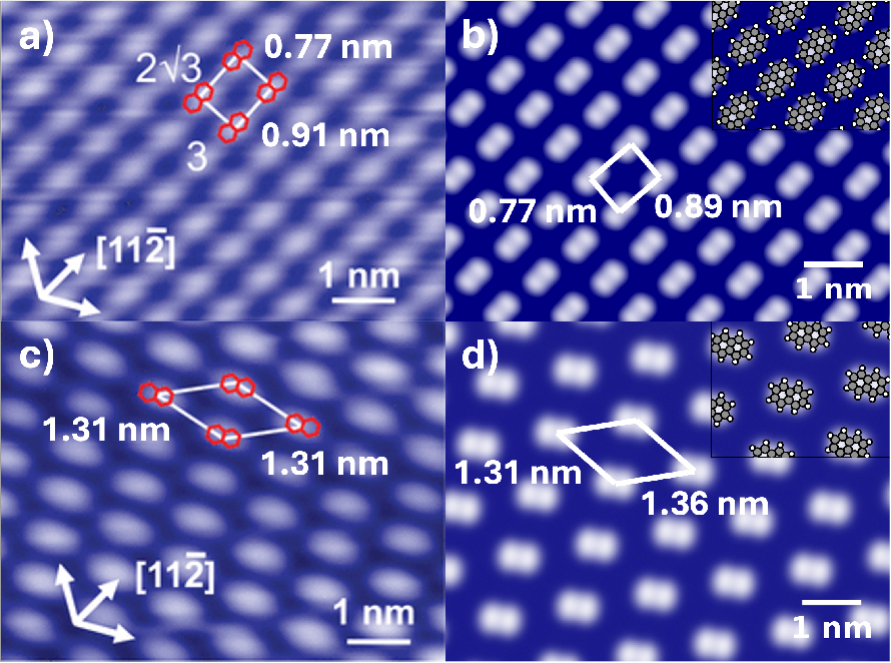
Comparison between simulated STM images of structures generated by Genarris Interfaces and the experimentally observed structures of the naphthalene/Cu(111) interface with *Z* = 1, reproduced with permission from ref 121 Copyright 2010 American Chemical Society. (a) Experimental STM image of the structure observed at *T* = 120 K. (b) Simulated STM image of the structure that most closely resembles the 120 K experimental structure, circled in pink in Figure 14a and shown in Figure 14b. (c) Experimental STM image of the structure observed at *T* = 140 K. (d) Simulated STM image of the structure that most closely resembles the 140 K experimental molecular arrangement, circled in orange in Figure 14a and shown in Figure 14c.

The STM image of the 140 K structure is shown in Figure 15c. The most stable structure with the same coverage is circled in green in Figure 14a and shown in Figure 14d. However, its γ angle (96°) differs from that of the experimental structure (139°). A candidate structure closely matching the 140 K phase is ranked as the most stable structure with a slightly lower coverage. This structure is highlighted in orange in Figure 14a and shown in panel e. Its simulated STM image is presented in Figure 15d, showing excellent agreement with experiment. An analysis of the adsorption sites of the most stable structures of naphthalene on Cu(111) is provided in the SI. We find that the long-bridge adsorption site on the Cu(111) surface is preferable, in agreement with ref 121. Thus, Genarris Interfaces successfully generated structures that closely resemble both of the observed structures of naphthalene on Cu(111) with *Z* = 1 near the experimental coverage values.

Figure 16 shows the interface area distributions of naphthalene film structures with *Z* = 6 generated by Genarris Interfaces after key steps of the structure generation and down-selection process. With six molecules per unit cell, all of the allowed layer groups have hexagonal symmetry. Therefore, all generated structures have γ = 120°, which is consistent with experiment.^121^ The raw pool of film structures (panel a) consists of 2053 structures with interface areas ranging from 0.74 nm^2^ to 6.17 nm^2^. 402 (19.6%) of these structures are in the interface area bin of the experimental naphthalene/Cu(111) interface structure with *Z* = 6, which is indicated by the red dashed line at 4.43 nm^2^. After duplicate removal (panel b), 1379 structures remain in the pool, 264 (19.1%) of which are in the experimental area bin. The area distribution remains similar to that of the raw pool. After *k*-means clustering (panel c) the interface area distribution becomes more uniform and the experimental area bin becomes the most populated. Of the 96 remaining film structures, 15 (15.6%) are in the experimental area bin. These 96 structures proceed to surface matching and DFT relaxation.

**Figure 16:**
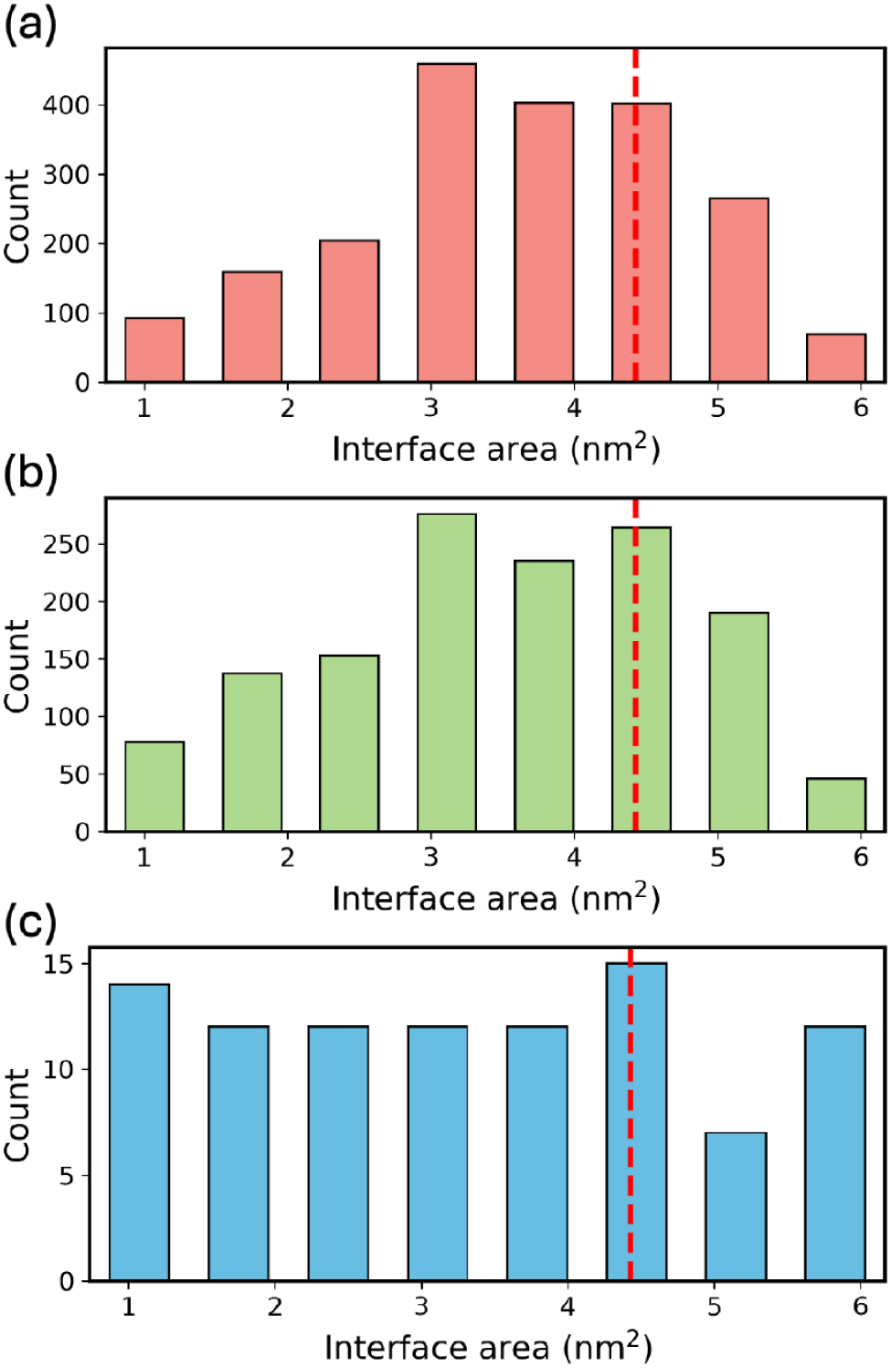
Interface area histograms of naphthalene film structures with *Z* = 6 obtained after key steps in the structure generation and down-selection workflow: (a) film slab construction, (b) duplicate removal, and (c) *k*-means clustering. The experimental interface area of 4.43 nm^2^ is indicated by the red dashed lines.

Figure 17a presents the adhesive energy as a function of coverage for the final relaxed naphthalene/Cu(111) interface structures with *Z* = 6. Owing to the high number of molecules per unit cell, multilayer structures are generated as the coverage increases, and stable planar monolayer configurations (colored in dark blue) are only possible at low coverage. The most stable structure overall, highlighted in cyan in panel a and shown in panel b, is a planar bilayer structure. The two layers have the same molecular arrangement with a lateral displacement in the *x*–*y* plane, and the molecules form a triangular packing motif within each layer. The least stable structure, highlighted in pink in panel a and shown in panel c, is a monolayer structure comprising triangles of molecules adsorbed upright on the short edge.

**Figure 17:**
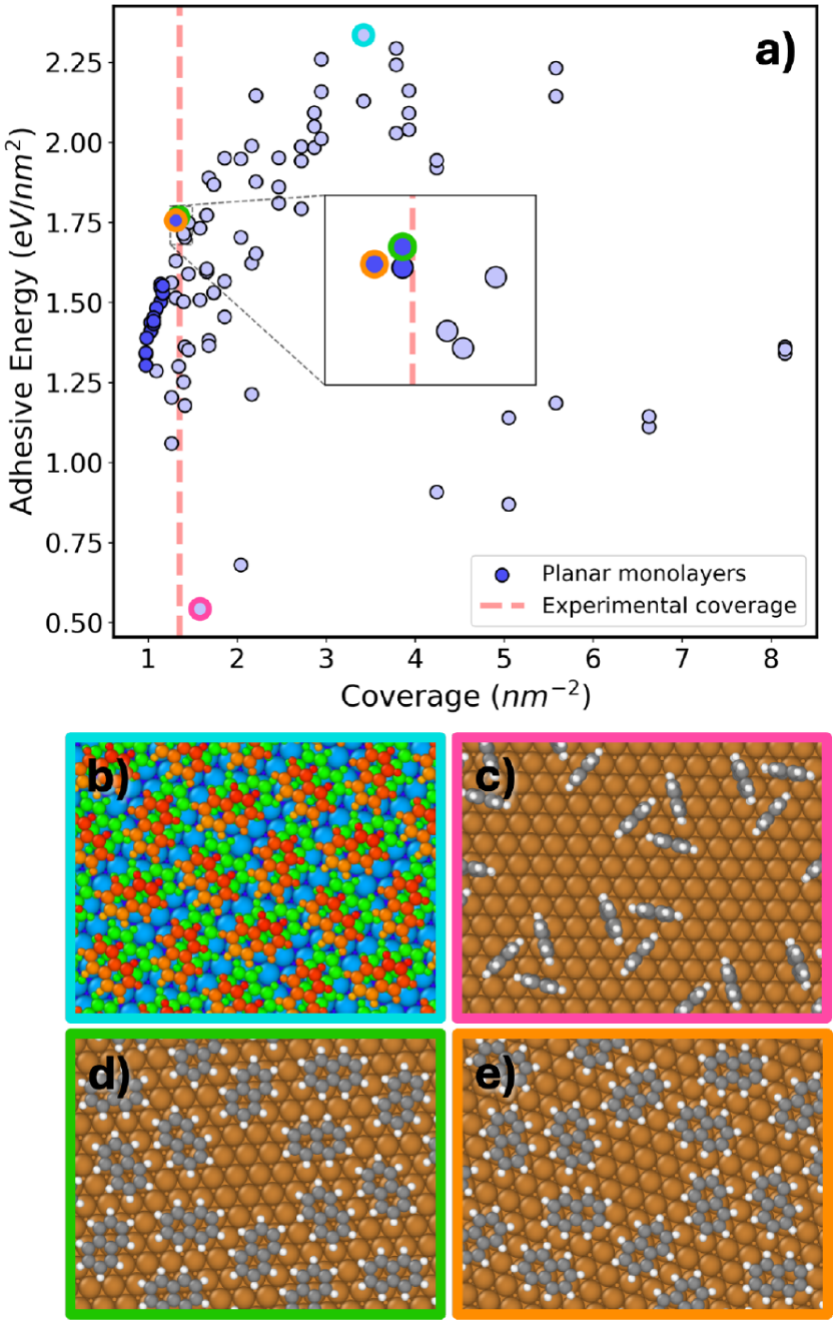
(a) Adhesive energy as a function of coverage for the final pool of relaxed naphthalene/Cu(111) structures with *Z* = 6. Planar monolayer structures are colored in dark blue. (b) The structure with the highest adhesive energy overall, adopting a bilayer structure, circled in cyan in panel a. The atoms are colored based on their height. (c) The least stable structure in the structure pool, adopting an upright adsorption mode, circled in pink in panel a. (d) The most stable structure with the experimental coverage, which resembles the experimental structure, circled in green in panel a. (e) The structure that most closely resembles the experimental structure, circled in orange in panel a.

The most stable structures around the experimental coverage (indicated by the dashed line) are highlighted in green and orange in panel a and shown in panels d and e, respectively. Both structures are very close in energy, have similar lattice parameters, and are characterized by a similar packing motif comprising groups of six molecules in a circular formation that resembles an aperture. The third member of this cluster has a similar unit cell but does not have the aperture packing motif, as shown in the SI. Simulated STM images of the two most stable structures are compared to experiment in Figure 18. The experimental STM images of the *Z* = 6 phase, shown in panels a and b, reveal two domains, in which the molecules form similar aperture arrangements but with opposite chirality.^121^ The structure highlighted in orange in Figure 17a, whose simulated STM image is shown in panel c, most closely resembles the molecular arrangement of the experimental structure. The structure highlighted in green in [Fig fig17]a, whose simulated STM image is shown in panel d, is the most stable structure closest to the experimental coverage. This structure has a very similar packing motif to the one shown in panel c, but with the molecules at a slightly different rotation, and its lattice parameters are somewhat closer to experiment. The presence of structures with similar packing motifs that are very close in energy is consistent with the experimental observation of coexisting domains with similar or symmetrically equivalent molecular arrangements. We also note that the molecules in both structures adopt the long-bridge adsorption mode, in agreement with ref 121. A previous study that used the SAMPLE code to generate structures of naphthalene on Cu(111) states that close matches to the three experimentally observed phases were produced but not ranked as the global minimum, although the matched structures are not shown therein.^81^

**Figure 18:**
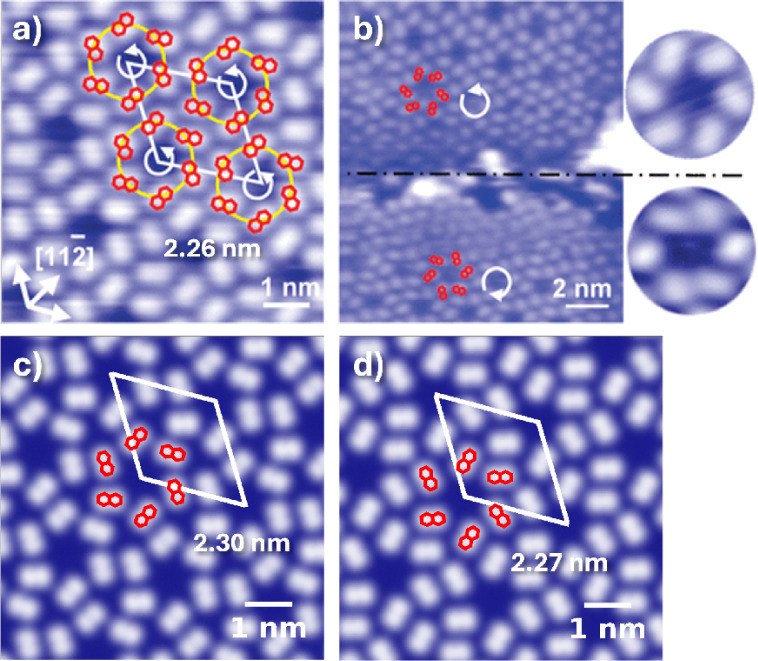
Comparison between simulated STM images of structures generated by Genarris Interfaces and the experimentally observed structures of the naphthalene/Cu(111) interface with *Z* = 6, reproduced with permission from ref 121 Copyright 2010 American Chemical Society. (a) Experimental STM image of the adsorption configuration of naphthalene/Cu(111) with *Z* = 6. (b) Large area STM image showing different adsorption modes in two domains separated by a monatomic step. (c)–(d) Simulated STM images of structures generated by Genarris: (c) The structure shown in Figure 17b, circled in orange in Figure 17a. (d) The structure shown in Figure 17c, circled in green in Figure 17a.

A discussion of the electronic structure of naphthalene/Cu(111) is presented in the SI. Briefly, UPS measurements of a naphthalene film on Cu(111) have been reported in the SI of ref 156. However, the UPS data are in an energy range, where there are only contributions from Cu states and no contributions from the naphthalene film. Therefore, it is difficult to make a detailed comparison between the DOS of the interface structures generated here and the UPS data. It is reported in ref 156 that naphthalene adsorption induces a downward shift of the Fermi level by 0.19 eV. As shown in the SI, this shift is best reproduced by the HSE DOS of the candidate structure with *Z* = 1 at 120 K.

## 5 Conclusion

In summary, we have introduced Genarris Interfaces, an open-source package for structure prediction of organic/inorganic interfaces that targets the challenge of efficiently exploring the vast configurational space of molecular films on crystalline substrates. Genarris Interfaces enforces commensurability via epitaxy matrices and generates candidate film unit cells across all layer groups compatible with the substrate lattice and the number of molecules per unit cell. Genarris does not make any assumptions regarding the adsorption site(s) or the adsorption mode(s) of the molecules on the surface. Thus, it is able to generate film structures with diverse packing arrangements, including monolayer and multilayer structures with varying surface coverage. After structure generation, a sequence of down-selections steps is executed to reduce the computational load. Duplicate removal is performed, followed by *k*-means clustering, and selection of representative structures from each cluster. Subsequently, interface structures are constructed and pre-optimized by performing “surface-matching” using Bayesian optimization with a fast geometric score function. Finally, full relaxation is performed using dispersion-inclusive DFT geometries, after which the adhesive energy is evaluated as the metric for stability ranking.

We demonstrated the application of Genarris Interfaces for three representative molecule/metal systems: PTCDA/Ag(111), TCNE/Au(111), and naphthalene/Cu(111). In all cases, Genarris generated structures that closely match experimental STM images in terms of the surface coverage, the unit cell dimensions, and the molecular packing arrangements. In particular, for naphthalene/Cu(111) Genarris generated all three “surface polymorph” structures observed under different temperature and coverage conditions. In all cases, the best-matched structures generated by Genarris are ranked as the most stable or among the top few in their coverage bins by PBE+TS^surf^. The DOS of the interface structures generated by Genarris, calculated using the HSE hybrid functional, are in good agreement with available UPS and STS data, although we note that it may be difficult to resolve the spectral signatures of different film structures because they are overshadowed by the prominent contributions of the metal surface.

Based on the results presented here, we conclude that Genarris Interfaces is a useful package for predicting the structure of molecular films on top of surfaces. The electronic properties of the resulting structures can then be calculated to assess their suitability for target applications and/or to interpret spectroscopy experiments. Future improvements to Genarris Interfaces may include using machine-learned interatomic potentials (MLIPs)^157^ to replace the geometric score function in the surface matching step and possibly to replace DFT for relaxation and ranking. This would require rigorous benchmarks of the performance of MLIPs for these tasks. In addition, we plan to implement workflows for structure prediction of interfaces involving heteromolecular films and films of flexible molecules that may change conformation upon adsorption. We also note that Genarris is not restricted to films on metal surfaces, although it has yet to be tested for other types of inorganic and organic substrates. We envision Genarris Interfaces being used to explore the structure and properties of candidate organic/inorganic interfaces and help select the most promising candidates for experimental growth and characterization. In addition, Genarris Interfaces may be used to generate DFT data sets for training MLIPs.

## Supplementary Material



## Data Availability

Genarris Interfaces is available on GitHub (https://github.com/haoran-ni/Genarris-Interfaces) and through the website (https://www.noamarom.com/software/download/) under the BSD-3-Clause license. The CubeSTM code for STM simulations is available on GitHub (https://github.com/haoran-ni/CubeSTM). All interface structures generated here, including all intermediate structures from the workflow, are available through Zenodo at (https://zenodo.org/records/18463810) with DOI 10.5281/zenodo.18463810.

## Notes

Description of CubeSTM; input parameters used for simulated STM images; convergence tests for DFT settings: *k*-point grid; number of substrate layers; amount of vacuum space; FHI-aims numerical settings; basis sets; effect of the DFT functional and dispersion method on the stability ranking of PTCDA/Ag(111) structures; effect of relaxation convergence settings on upright vs planar structures of TCNE/Au(111); effect of the DFT functional and dispersion method on the stability ranking of TCNE/Au(111); DOS of TCNE film structures with different coverage and adsorption modes; visualizations of additional generated structures of naphthalene/Cu(111) with *Z* = 1 and *Z* = 6; DOS of naphthalene/Cu(111) structures compared to UPS (PDF)
