# Supplementary material for: Structure Prediction of Organic/Inorganic Interfaces with Genarris
Source: J Chem Theory Comput. 2026 Apr 23;22(9):4835–53. doi: 10.1021/acs.jctc.6c00235 (PMC13173537; doi:10.1021/acs.jctc.6c00235)
Supplement: Supplementary file 1 [file ct6c00235_si_001.pdf]

# Structure Prediction of Organic/Inorganic Interfaces with Genarris

## Supporting Information

Haoran Ni[1], Kevin Larkin[1], Wen Wen[2], Saeed Moayedpour[2], Rithwik Tom[3], Imanuel Bier[1], Derek Dardzinski[1], and Noa Marom[1,2,3]

[1]Department of Materials Science and Engineering, Carnegie Mellon University, Pittsburgh, PA 15213

[2]Department of Chemistry, Carnegie Mellon University, Pittsburgh, PA 15213

[3]Department of Physics, Carnegie Mellon University, Pittsburgh, PA 15213

March 26, 2026

Email address: nmarom@andrew.cmu.edu

## Contents

**1 STM simulations** **S2**
- 1.1 CubeSTM . . . . . S2
- 1.2 Parameters used for STM simulations . . . . . S4

**2 Convergence tests** **S9**
- 2.1 *k*-point grid convergence . . . . . S10
- 2.2 Substrate layers convergence . . . . . S12
- 2.3 Vacuum space convergence . . . . . S14
- 2.4 Numerical settings convergence . . . . . S16

**3 Additional results for PTCDA/Ag** **S17**

**4 Additional results for TCNE/Au** **S17**

**5 Additional results for Naphthalene/Cu(111)** **S19**

# 1 STM simulations

## 1.1 CubeSTM

The simulated STM images in the main text were generated using the CubeSTM code. Our implementation extends the Tersoff-Hamann[1] method by considering *non-local tunneling*, *screening effects*, and *localization effects* to create improved STM simulations. The Tersoff-Hamann method is commonly used for simulations of STM images from partial charge files produced by DFT codes. It is assumed that the tunneling current is proportional to the local density of states (LDOS) at the position of the tip and that the potential of the STM tip is spherically symmetric. As a result, under the constant current mode, the STM topography can be interpreted as the isosurface of the LDOS. However, STM images simulated using the Tersoff-Hamann method do not always agree well with experimental data. The edges may appear too sharp and in some cases there are discrepancies in the shape of the electron densities. To address these deficiencies, we developed CubeSTM, which can be better understood under the constant height mode. We consider the following:

- *Non-local tunneling*: All electron densities within a confined neighborhood near the STM tip may contribute to the tunneling current with a probability factor of the form:

$$P_{non-local}(r) = \exp{(-\alpha \cdot r)}, \tag{1}$$

where $r$ is the distance from a point to the position of the STM tip, and $\alpha$ is a user-defined parameter. This modification effectively softens the edges of the electron densities, creating a blurry effect similar to experimental STM images.

- *Screening effect*: The higher the cumulative electron density between a point and the STM tip, the less likely that point is to contribute to the tunneling current, owing to screening. This factor is written as:

$$P_{screening} = \exp{(-\beta \cdot \int \rho(\mathbf{r}) d\mathbf{r})}, \tag{2}$$

where $\int \rho(\mathbf{r}) d\mathbf{r}$ integrates over the local electron density between the current point and the position of the STM tip, and $\beta$ is a user-defined parameter.

- *Localization effect*: The electron density close to the nuclei may be higher than the electron density in the valence region by orders of magnitude. As a result, the localized, strongly bound electrons close to the nuclei may contribute disproportionately to the simulated tunneling current. To mitigate this, we introduce the assumption that the higher the electron density at one point, the less likely it is to contributes to the tunneling current. This is expressed by the factor

$$P_{localization} = \exp{(-\gamma \cdot \rho(\mathbf{r}) / \rho_{max})}, \tag{3}$$

where $\rho(\mathbf{r})$ is the electron density at point $\mathbf{r}$, $\rho_{max}$ is the maximum local electron density in the system, and $\gamma$ is a user-defined parameter.

We note that the mathematical forms of the above factors were chosen empirically, owing to the lack of simple analytical expressions for such effects. In typical implementations of STM simulations, the electron densities are integrated only along the $z$ direction for simplicity, not along the direction to the STM tip. And by $\rho(\mathbf{r})$ and *local electron density*, we are referring to the charge density integrated from the Fermi level to the applied bias voltage.

The final expression for the tunneling current is thus

$$I(\mathbf{r}) = \int_{<r_{cut}} P_{non-local} \cdot P_{screening} \cdot P_{localization} \cdot \rho(\mathbf{r}')d\mathbf{r}', \tag{4}$$

where $\mathbf{r}$ is the position of the STM tip, and the integration runs over the neighborhood whose cutoff distance $r_{cut}$ is determined by the user input.

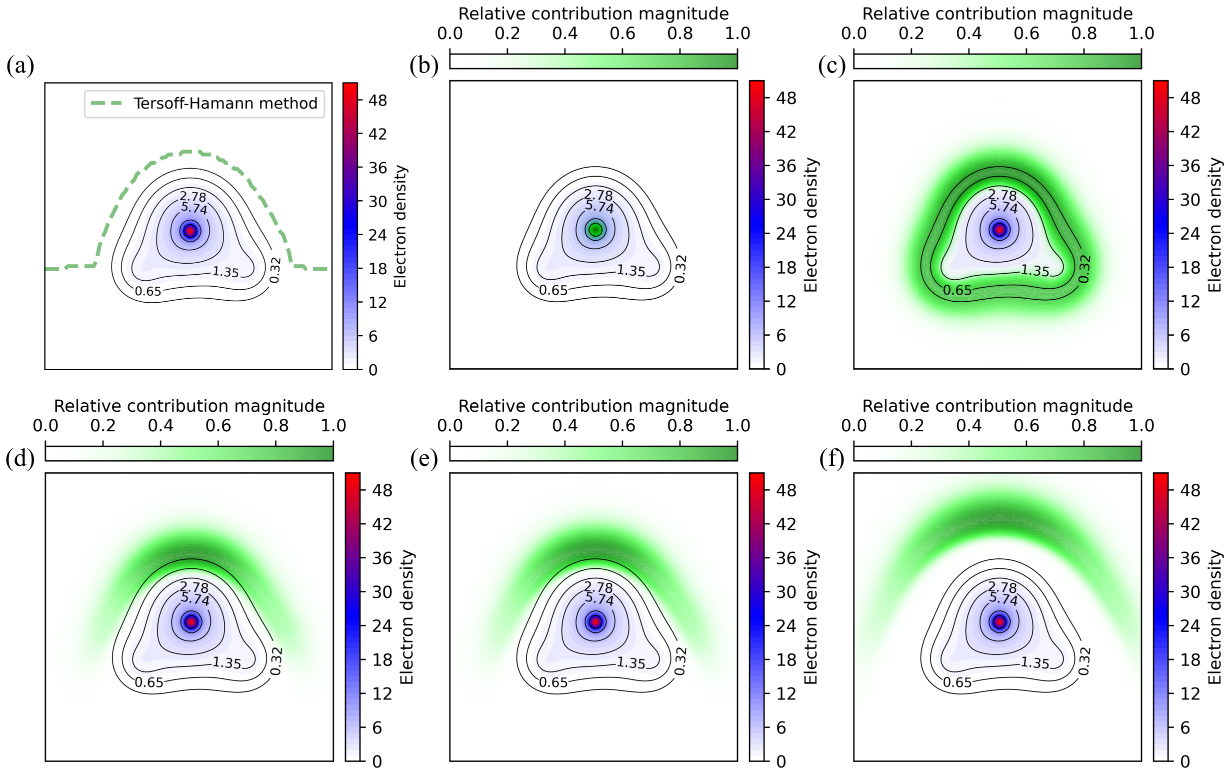


**Figure S1:** Illustration of relative contributions of electron densities to the tunneling current under different parameters in CubeSTM. The visualized area is a cross section in the $x - z$ plane, where the STM bias voltage is applied along the $z$ direction, namely the vertical direction in this figure. (a) The Tersoff-Hamann isosurface. (b) Screening effect off, localization effect off. (c) Screening effect off, localization effect on. (d) Screening effect on, localization effect off. (e) Screening effect on, localization effect on. (f) Screening effect magnified, localization effect magnified.

To illustrate how CubeSTM works, we take the electron densities of an isolated water molecule as an example, as shown in Fig. S1. Panel (a) shows the isosurface plotted using the Tersoff-Hamann method. Panel (b) shows that without the screening effect and the localization effect, most contributions to the tunneling current come from the inner-shell electrons close to the nuclei, where the electron densities are several orders higher than outer-shell electrons. In panel (c), the localization effect is applied but not the screening effect. In this case the inner-shell electrons stay confined, yet we still see contributions from electrons even below the nuclei, which is not correct. In panel (d), the screening effect is applied but not the localization effect. We see that the screening effect alone includes similar effects as the localization effect, where inner-shell

electrons are confined. Both the screening effect and the localization effect are applied in panel (e), which demonstrates the contributions of the electron densities to the tunneling current in CubeSTM have a similar distribution of an isosurface, but slightly delocalized to create similar effects as observed in experimental STM images. In the last panel (f), we greatly increase the strength of both the screening effect and the localization effect, which effectively visualizes the outer-shell electron distribution.

## 1.2 Parameters used for STM simulations

The following CubeSTM parameters were used to generate the simulated STM images shown:

| STM simulation | Parameters | |
|---|---|---|
| 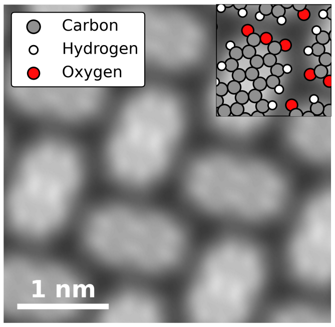 | neighbourhood | 3 |
| | z_decay | 0.8 |
| | xy_decay | 0.8 |
| | screening | 5 |
| | localization | 5 |
| | v_min | 0.1 |
| | v_max | 1.1 |
| | sigma | 3 |
| 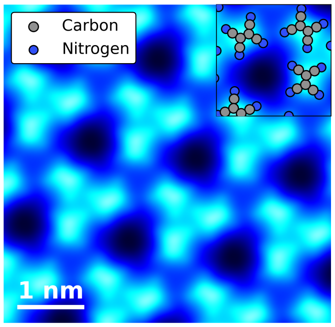 | neighbourhood | 8 |
| | z_decay | 0.2 |
| | xy_decay | 0.5 |
| | screening | 10 |
| | localization | 10 |
| | v_min | 0.55 |
| | v_max | 1.07 |
| | sigma | 3 |

| STM simulation | Parameters | |
|---|---|---|
| 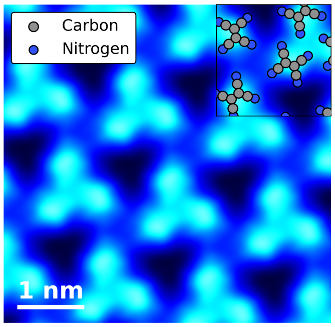 | neighbourhood | 8 |
| | z_decay | 0.2 |
| | xy_decay | 0.4 |
| | screening | 10 |
| | localization | 10 |
| | v_min | 0.78 |
| | v_max | 1.04 |
| | sigma | 3 |
| 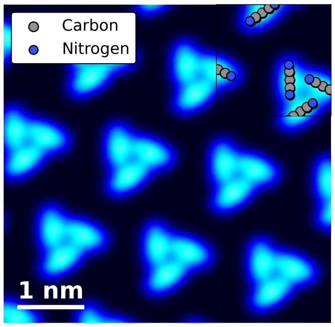 | neighbourhood | 2.6 |
| | z_decay | 0.3 |
| | xy_decay | 0.8 |
| | screening | 10 |
| | localization | 10 |
| | v_min | 0.0 |
| | v_max | 1.2 |
| | sigma | 3 |
| 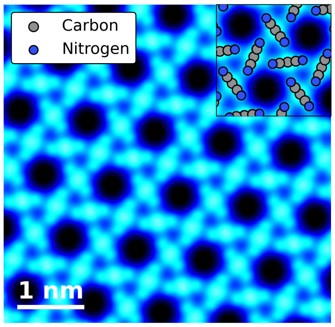 | neighbourhood | 2.5 |
| | z_decay | 0.5 |
| | xy_decay | 1 |
| | screening | 10 |
| | localization | 10 |
| | v_min | 0.1 |
| | v_max | 1.15 |
| | sigma | 3 |

| STM simulation | Parameters | |
|---|---|---|
| 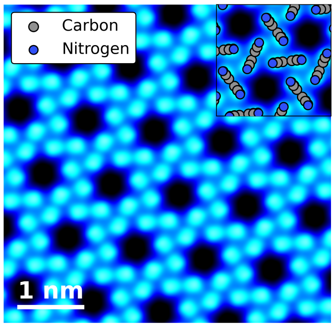  | neighbourhood | 2.2 |
| | z_decay | 0.5 |
| | xy_decay | 1 |
| | screening | 10 |
| | localization | 5 |
| | v_min | 0.1 |
| | v_max | 1.15 |
| | sigma | 3 |
| 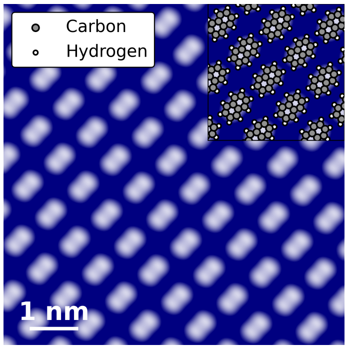  | neighbourhood | 2 |
| | z_decay | 1 |
| | xy_decay | 0.8 |
| | screening | 5 |
| | localization | 5 |
| | v_min | 0.45 |
| | v_max | 1.1 |
| | sigma | 3 |
| 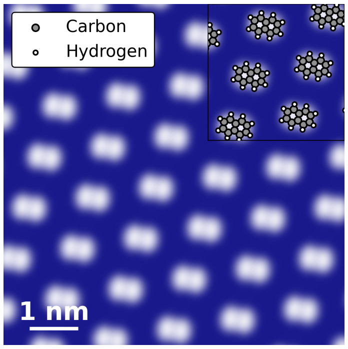  | neighbourhood | 2 |
| | z_decay | 1 |
| | xy_decay | 0.8 |
| | screening | 5 |
| | localization | 5 |
| | v_min | 0.01 |
| | v_max | 1.1 |
| | sigma | 3 |

<table>
<tr><th>STM simulation</th><th colspan="2">Parameters</th></tr>
<tr><td rowspan="8">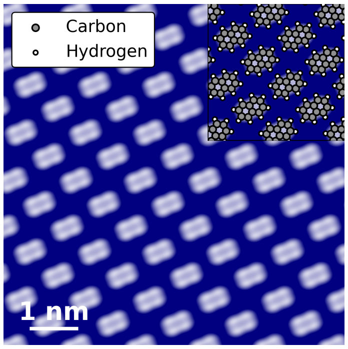
</td><td>neighbourhood</td><td>2</td></tr>
<tr><td>z_decay</td><td>1</td></tr>
<tr><td>xy_decay</td><td>0.8</td></tr>
<tr><td>screening</td><td>5</td></tr>
<tr><td>localization</td><td>5</td></tr>
<tr><td>v_min</td><td>0.45</td></tr>
<tr><td>v_max</td><td>1.1</td></tr>
<tr><td>sigma</td><td>3</td></tr>
<tr><td rowspan="8">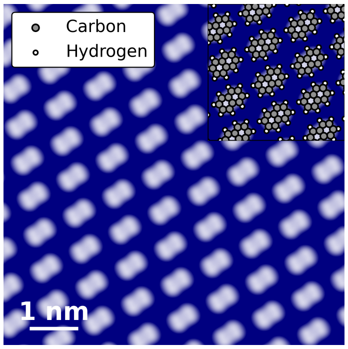
</td><td>neighbourhood</td><td>2</td></tr>
<tr><td>z_decay</td><td>1</td></tr>
<tr><td>xy_decay</td><td>0.8</td></tr>
<tr><td>screening</td><td>5</td></tr>
<tr><td>localization</td><td>5</td></tr>
<tr><td>v_min</td><td>0.45</td></tr>
<tr><td>v_max</td><td>1.1</td></tr>
<tr><td>sigma</td><td>3</td></tr>
<tr><td rowspan="8">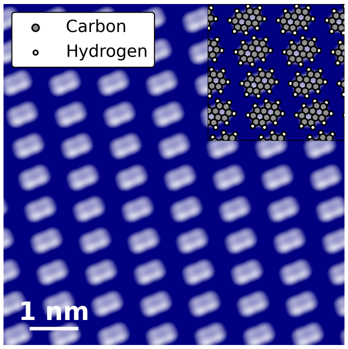
</td><td>neighbourhood</td><td>2</td></tr>
<tr><td>z_decay</td><td>1</td></tr>
<tr><td>xy_decay</td><td>0.8</td></tr>
<tr><td>screening</td><td>5</td></tr>
<tr><td>localization</td><td>5</td></tr>
<tr><td>v_min</td><td>0.45</td></tr>
<tr><td>v_max</td><td>1.1</td></tr>
<tr><td>sigma</td><td>3</td></tr>
</table>

| STM simulation | Parameters | |
|---|---|---|
| 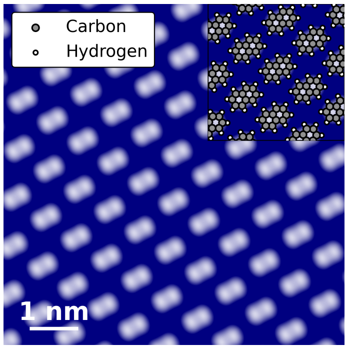 | neighbourhood | 2 |
| | z_decay | 1 |
| | xy_decay | 0.8 |
| | screening | 5 |
| | localization | 5 |
| | v_min | 0.45 |
| | v_max | 1.1 |
| | sigma | 3 |
| 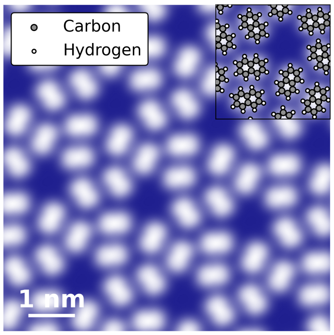 | neighbourhood | 3 |
| | z_decay | 1 |
| | xy_decay | 0.8 |
| | screening | 10 |
| | localization | 5 |
| | v_min | 0 |
| | v_max | 1 |
| | sigma | 2 |
| 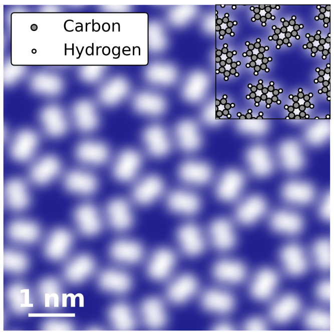 | neighbourhood | 3 |
| | z_decay | 1 |
| | xy_decay | 0.8 |
| | screening | 10 |
| | localization | 5 |
| | v_min | 0 |
| | v_max | 1 |
| | sigma | 2 |

| STM simulation | Parameters | |
|---|---|---|
| 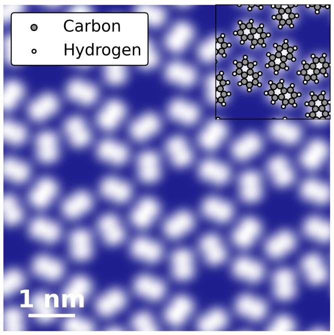 | neighbourhood | 2.5 |
| | z_decay | 1 |
| | xy_decay | 0.8 |
| | screening | 10 |
| | localization | 5 |
| | v_min | 0 |
| | v_max | 1 |
| | sigma | 2 |

## 2 Convergence tests

Convergence tests were performed to determine the optimal values of the $k$-point grid, the number of substrate layers, the total vacuum space, and the numerical settings and basis sets of FHI-aims. All calculations were conducted using the PBE[2] functional with the $TS^{surf}$[3] dispersion correction. Unless otherwise stated, the following settings were used: A k-point grid of $4 \times 4 \times 1$, 3 substrate layers, a total vacuum space of 60 Å, and light numerical settings with tier 1 basis sets.

The convergence was determined based on the relative total energy ranking of representative interface structures generated by Genarris (labeled by the structure ID generated by the code). The structures in each convergence test are selected from the surface matching structure pool (prior to DFT relaxation) with the same number of atoms, so that their total energies are comparable. The structures may have a different coverage. In each case, if the ranking of the structures remains stable, then the parameters are deemed converged. We find that the substrate thickness and choice of numerical settings have the most significant impact on the relative energies of interface structures.

## 2.1 $k$-point grid convergence

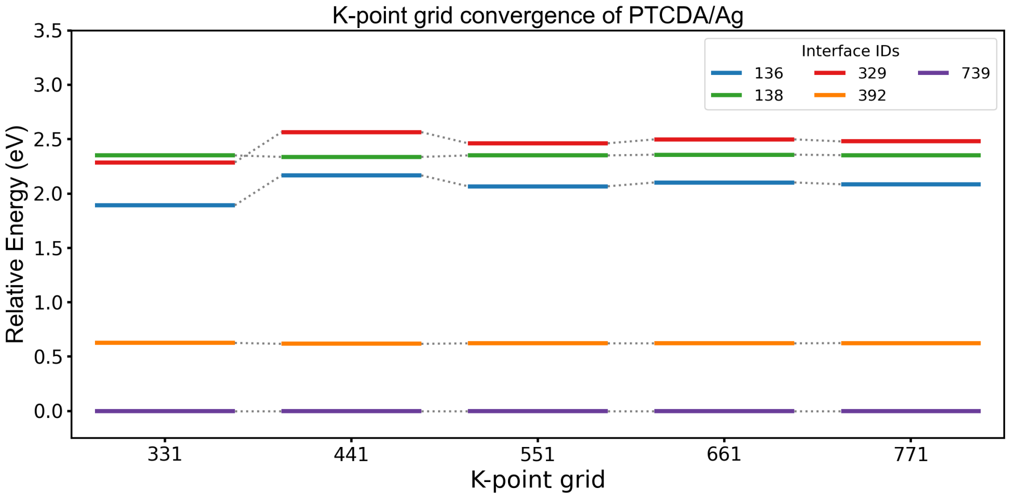


**Figure S2:** $k$-point grid convergence of PTCDA/Ag. The ranking is converged at $4 \times 4 \times 1$.

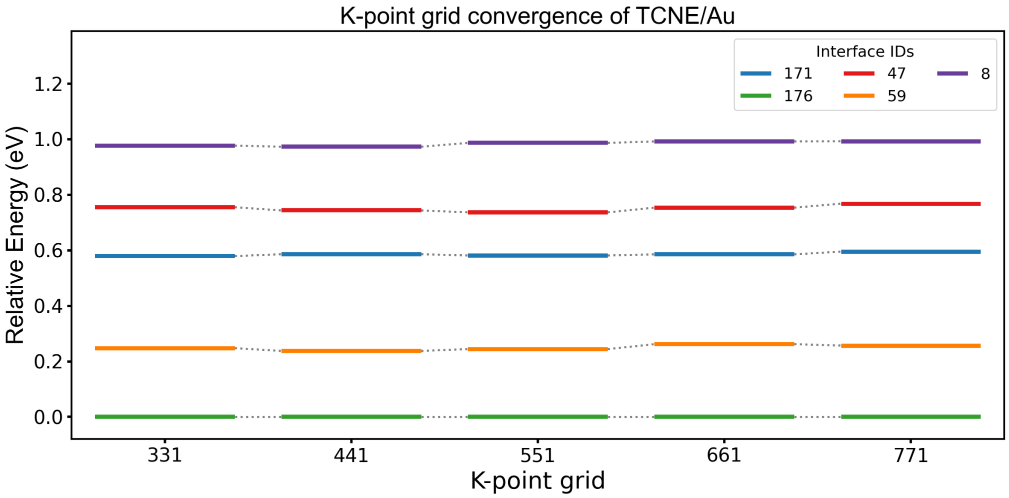


**Figure S3:** $k$-point grid convergence of TCNE/Au. The ranking is converged at $3 \times 3 \times 1$.

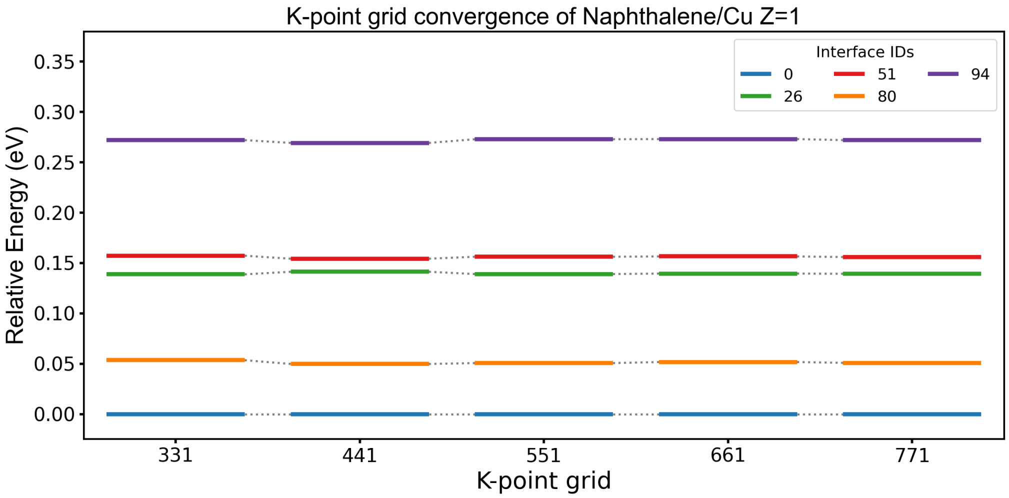


**Figure S4:** $k$-point grid convergence of naphthelene/Cu Z=1. The ranking is converged at $3 \times 3 \times 1$.

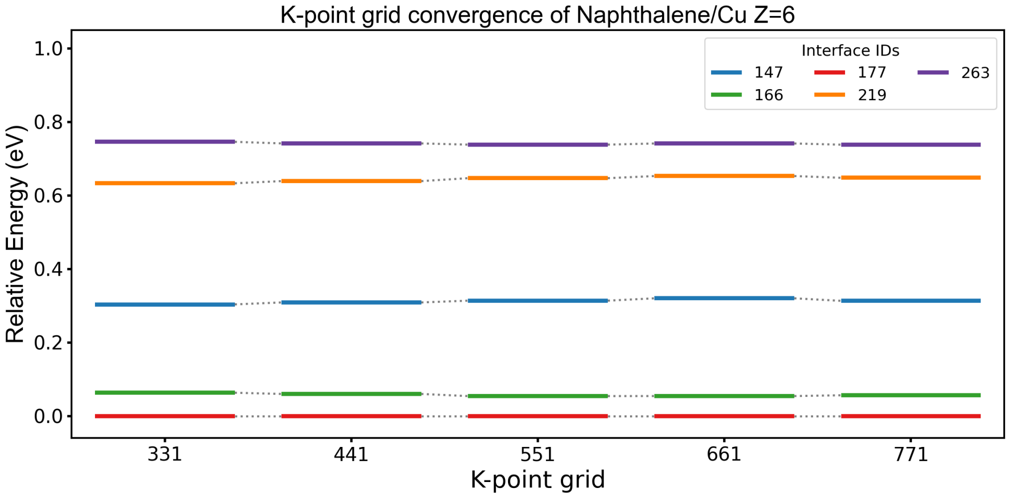


**Figure S5:** $k$-point grid convergence of naphthelene/Cu Z=6. The ranking is converged at $3 \times 3 \times 1$.

## 2.2 Substrate layers convergence

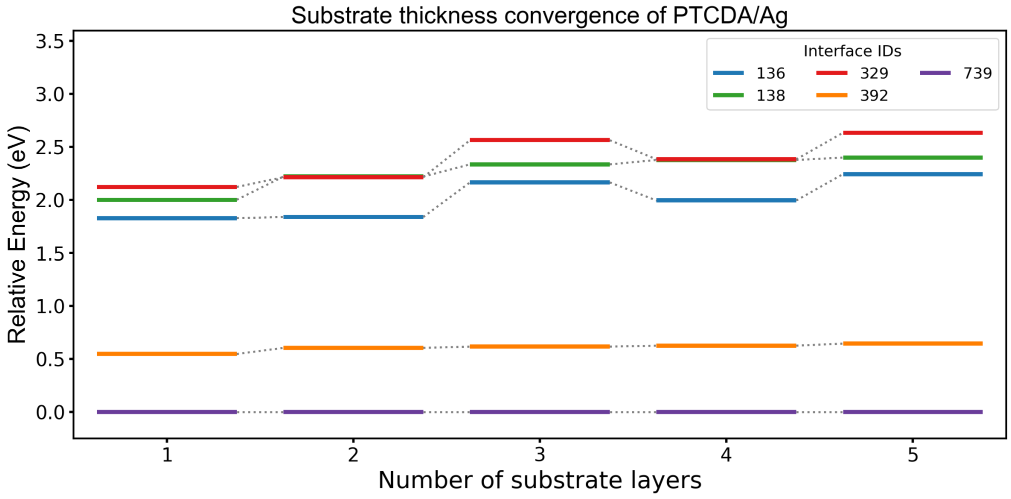


**Figure S6:** Substrate layers convergence of PTCDA/Ag. The ranking is converged at 3 layers.

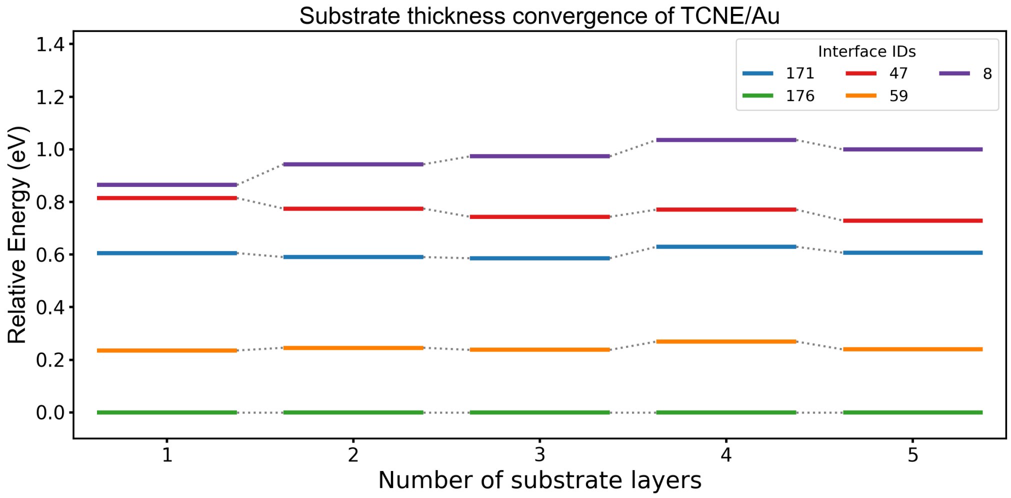


**Figure S7:** Substrate layers convergence of TCNE/Au. The ranking is converged at 2 layers.

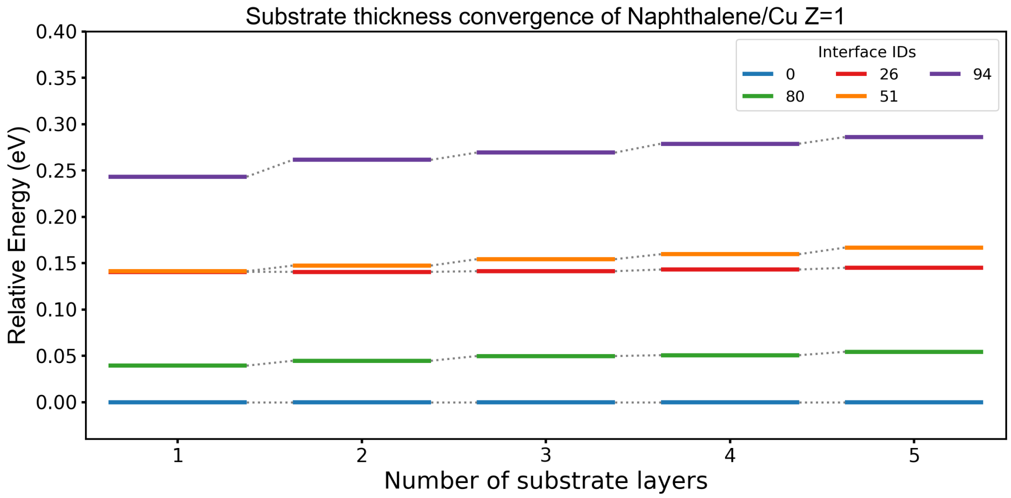


**Figure S8:** Substrate layers convergence of naphthalene/Cu Z=1. The ranking is converged at 2 layers.

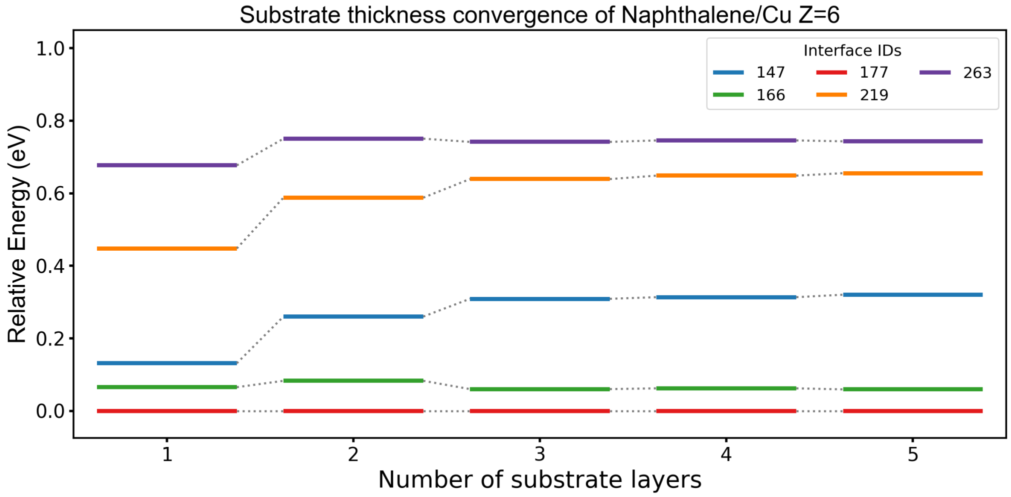


**Figure S9:** Substrate layers convergence of naphthalene/Cu Z=6. The ranking is converged at 1 layer.

## 2.3 Vacuum space convergence

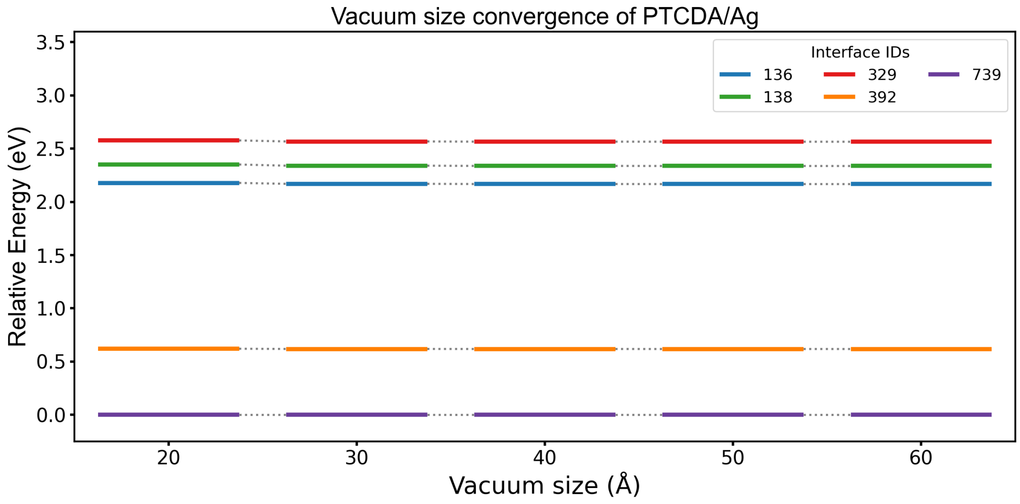


**Figure S10:** Vacuum space convergence of PTCDA/Ag. The ranking is converged at 20 Å of vacuum.

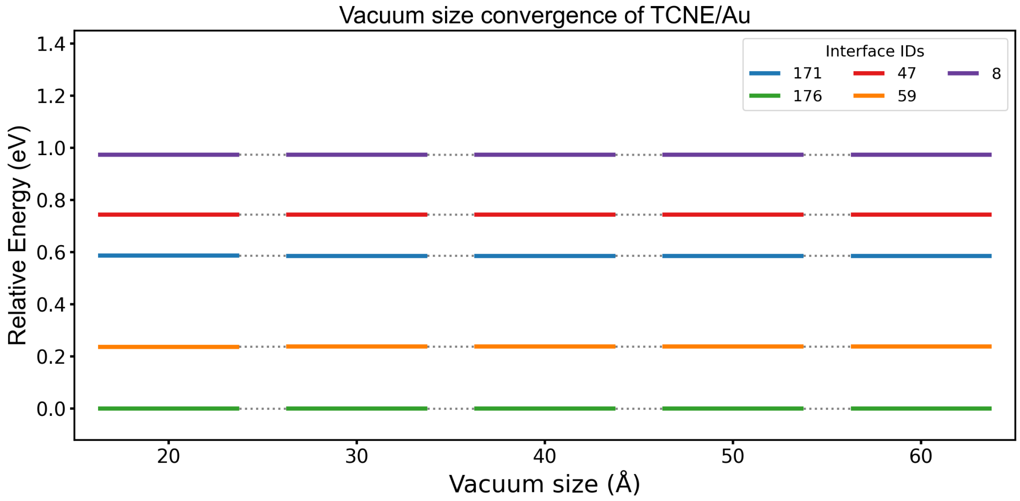


**Figure S11:** Vacuum space convergence of TCNE/Au. The ranking is converged at 20 Å of vacuum.

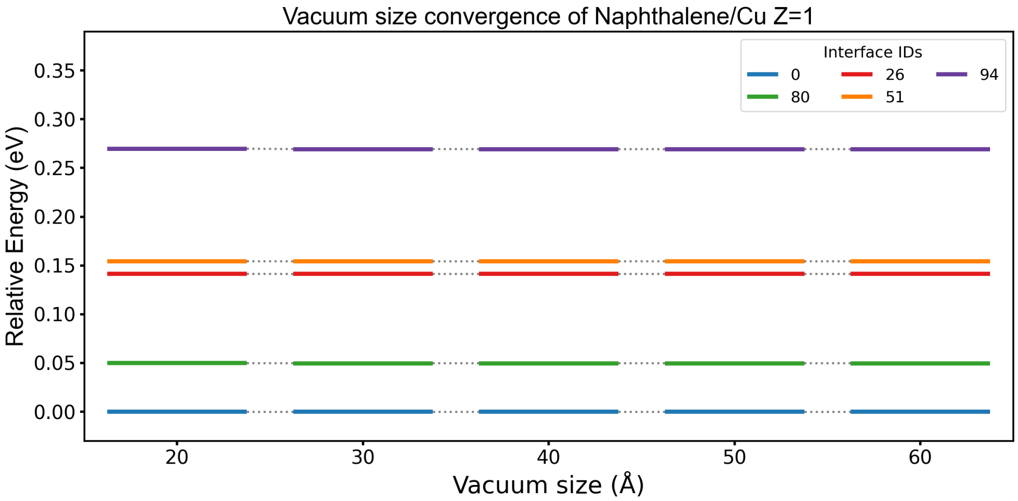


**Figure S12:** Vacuum space convergence of Naphthalene/Cu Z=1. The ranking is converged at 20 Å of vacuum.

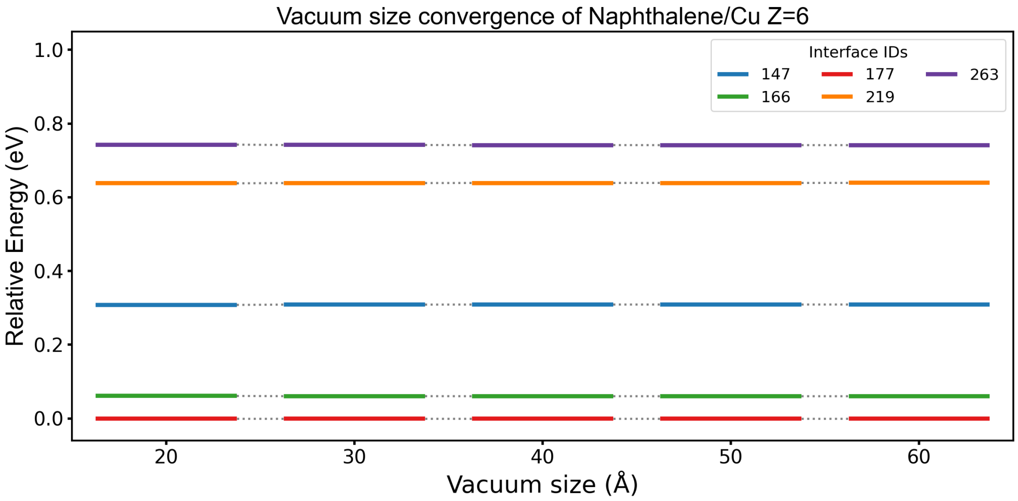


**Figure S13:** Vacuum space convergence of Naphthalene/Cu Z=6. The ranking is converged at 20 Å of vacuum.

## 2.4 Numerical settings convergence

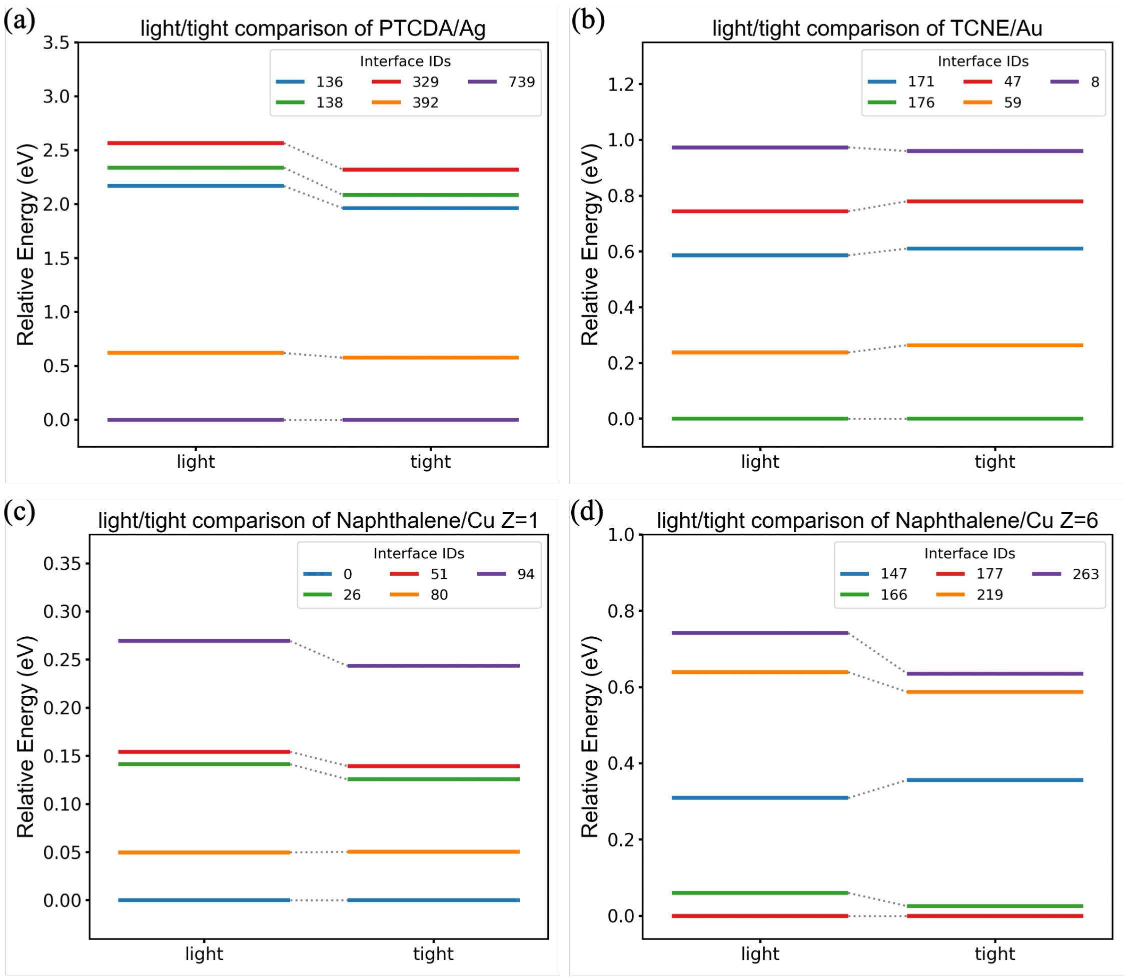


**Figure S14:** Convergence of numerical settings and basis sets for (a) PTCDA/Ag, (b) TCNE/Au, (c) naphthalene/Cu Z=1 and (d) naphthalene/Cu Z=6. Here we use *light* for light numerical settings with tier 1 basis sets, and *tight* for tight numerical settings with tier 2 basis sets. The rankings are converged with the light species settings and tier 1 basis sets.

# 3 Additional results for PTCDA/Ag

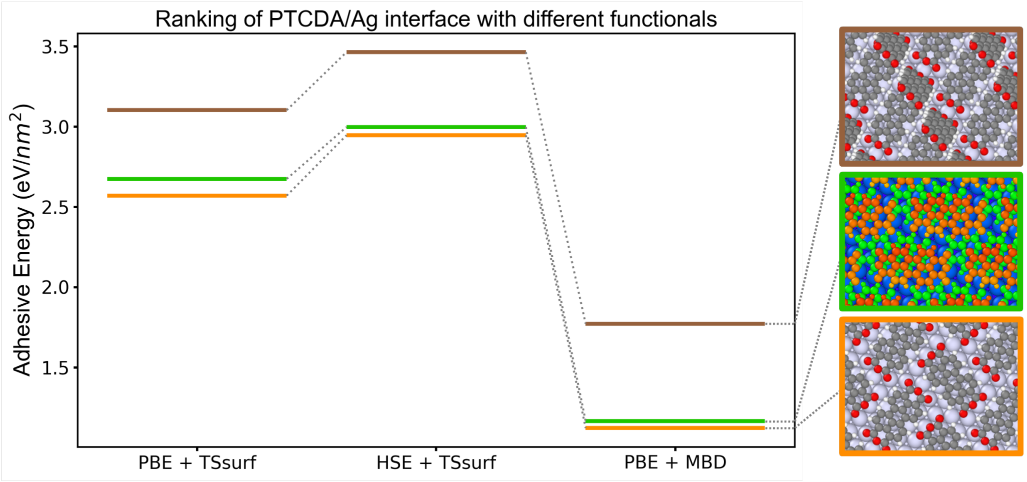


**Figure S15:** Comparison of the rankings of PTCDA/Ag interfaces with different DFT functionals and dispersion methods. The structure ranked as the most stable overall (circled in brown here and in Figure 6 in the main text) is compared to the second most stable bilayer structure with the same coverage (circled in green here and in Figure 6 in the main text) and to the best matched structure, ranked as the most stable in the experimental coverage bin (circled in orange here and in Figure 6 in the main text). The structure circled in brown is persistently ranked as the most stable using different DFT functionals and dispersion methods.

# 4 Additional results for TCNE/Au

In Fig. 10 in the main text, we notice that the majority of structures with TCNE molecules standing upright on the Au surface have the C = C bonds perpendicular to the surface, with only two structures having parallel C = C bonds at extremely high coverage. This result is inconsistent with the results from *SAMPLE*,[4] where a candidate structure with an upright parallel configuration at the experimental coverage is proposed.

Based on a detailed comparison of the DFT settings, we conclude that this discrepancy originates from the force convergence threshold used for relaxation. In the *SAMPLE* paper, a force convergence threshold of 0.1 $eV/Å$ was applied, which is relatively high and produces structures that are not fully relaxed. In comparison, we set the force convergence threshold for relaxation to 0.01 $eV/Å$, leading to the difference in the geometry of the relaxed structures. To demonstrate this, we selected two TCNE/Au interface structures generated by *Genarris Interfaces* with the C = C bonds parallel to the surface, and compared the geometries of the relaxed structures using a force convergence threshold of 0.1 $eV/Å$ and 0.01 $eV/Å$. We also considered different dispersion methods, as shown in Fig. S16. For both PBE + $TS^{surf}$ and PBE + MBD, all structures relaxed using a 0.1 $eV/Å$ force convergence threshold remain upright, whereas with a 0.01 $eV/Å$ threshold the fully relaxed structures do not remain upright, but have a planar or inclined configuration.

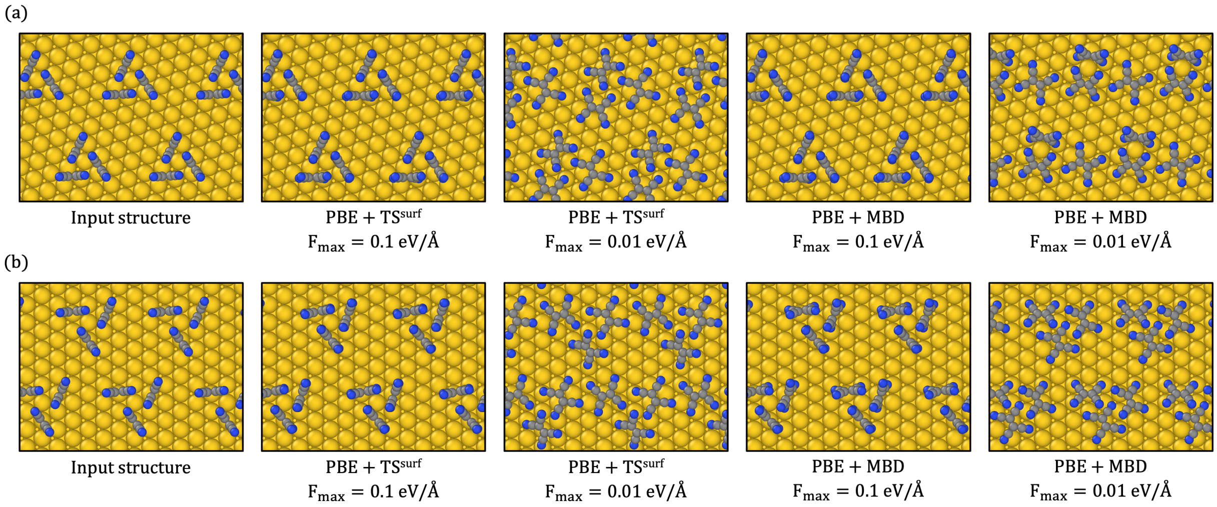


**Figure S16:** Relaxation results of TCNE/Au interfaces generated with an initial upright parallel configuration with different force convergence thresholds. Two different structures are shown in panels (a) and (b).

We further evaluated the effect of the choice of DFT functional and dispersion method on the relative stability of the planar vs. upright structures with the experimental coverage shown in Figures 10 and 11 in the main text. Figure S17 shows that the interface structure where the TCNE molecules are planar remains the most stable and the interface structure with upright TCNE molecules remains the least stable regardless of the choice of DFT functional and dispersion method.

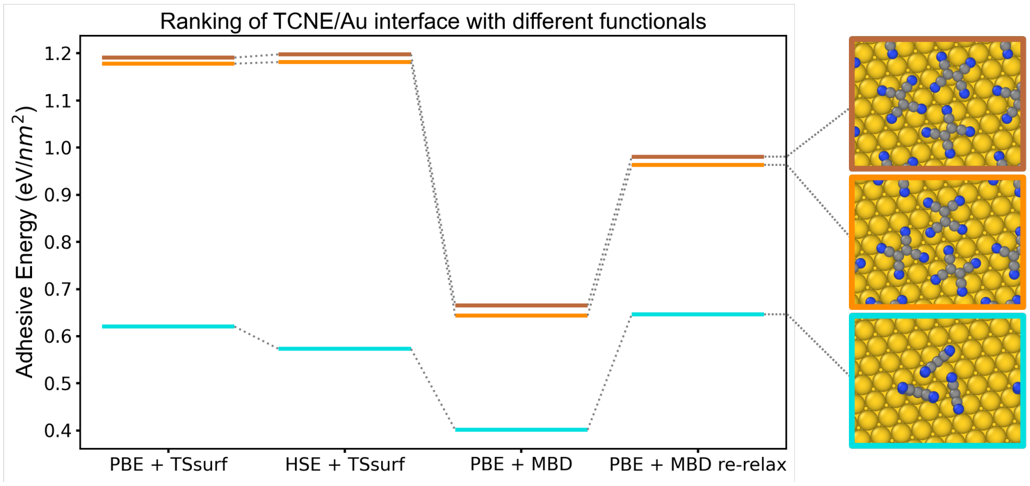


**Figure S17:** Comparison of the rankings of TCNE/Au interfaces with the experimental coverage with different DFT functionals and dispersion methods. The structures in the first three columns were relaxed with PBE + $TSs^{surf}$ and their adhesive energy was subsequently evaluated using the listed methods. The structures in the last column were relaxed with PBE + MBD, and their adhesive energies were subsequently evaluated using the same method.

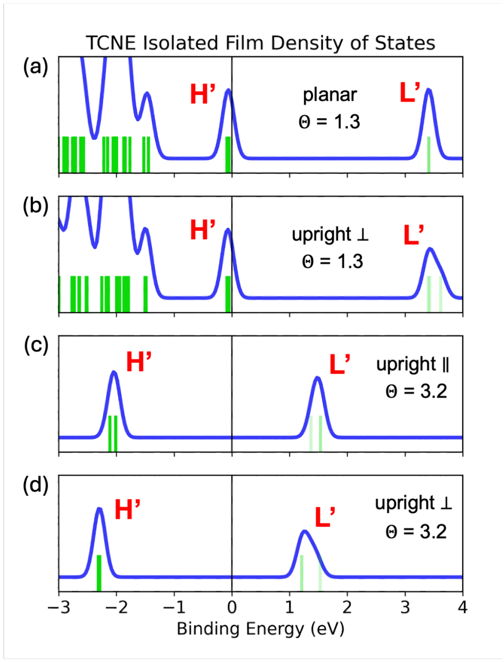


**Figure S18:** DOS of the isolated TCNE films. Discrete eigenstates are marked with green bars under the DOS curve, where occupied states and unoccupied states are colored with green and light green respectively. The structures from panels (a) to (d) here are the same structures as panels (b) to (e) in Figure 12 in the main text. The spacing of L'states for structures with planar adsorption modes is smaller than for structures with upright adsorption modes. Because this difference is observed for isolated TCNE films without the Au substrate, it is attributed to intermolecular interactions rather than molecule-metal interactions.

# 5 Additional results for Naphthalene/Cu(111)

The naphthalene/Cu(111) interface structures with higher adhesion energies than the best matched structure at 120K (circled with pink in Figure 14 in the main text) are shown in Fig. S19. These structures have a planar adsorption mode with parallelogram unit cells, each with slightly different cell parameters. We attribute the differences in their adhesion energy to the adsorption sites on top of the Cu(111) surface. In the two most stable structures (shown in panels a and b) the molecules adsorb on two hollow-hcp sites. In comparison, in the structure shown in panel c, one molecule adsorbs on a hollow-hcp site and another on a hollow-fcc site, and in the structure shown in panel the molecules adsorb on two hollow-fcc sites. The same adsorption preference is also found for $Z = 6$ structures. The three most stable structures near the experimental coverage are shown in Fig. S20. In the structure shown in panel a, all six molecules adsorb on two hollow-hcp sites. In the structure shown in panel b, three molecules adsorb on two hollow-hcp sites, and the other three adsorb on two hollow-fcc sites. In the structure shown in panel c, the adsorption sites are slightly displaced from the ideal sites.

The DOS of naphthalene/Cu(111) interface structures generated by Genarris, computed using the HSE functional are shown in Fig. S21. The DOS of the same Cu(111) slab (3 layers) is shown in the lower three panels for direct comparison. The DOS of the interface structure is shown in blue, and the projected DOS of

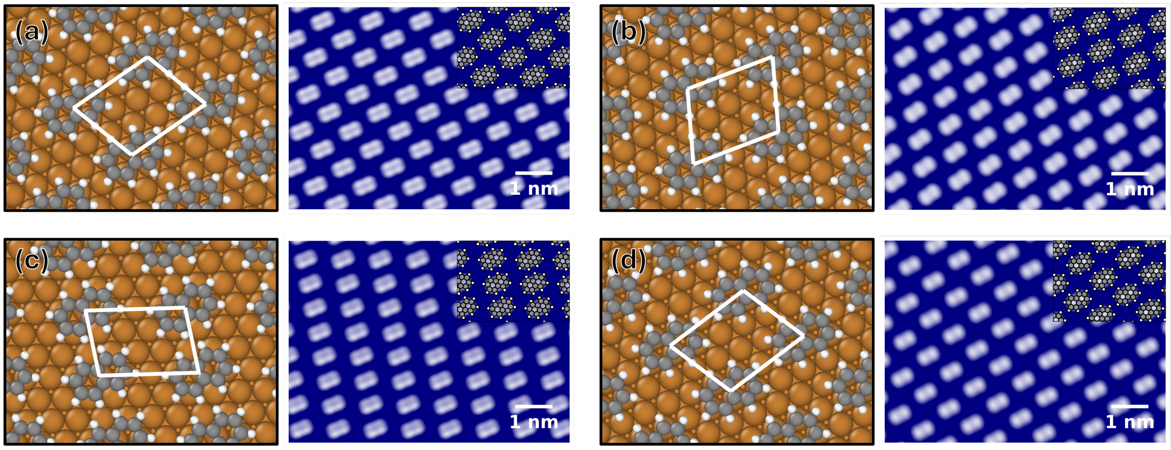


**Figure S19:** The four most stable structures of naphthalene on Cu(111) with Z=1 near the experimental coverage bin at 120 K, marked with blue dashed lines in Figure 14 in the main text. The structures in panels (a)-(d) are arranged in order of descending adhesion energy The simulated STM image of each structure is shown on the right.

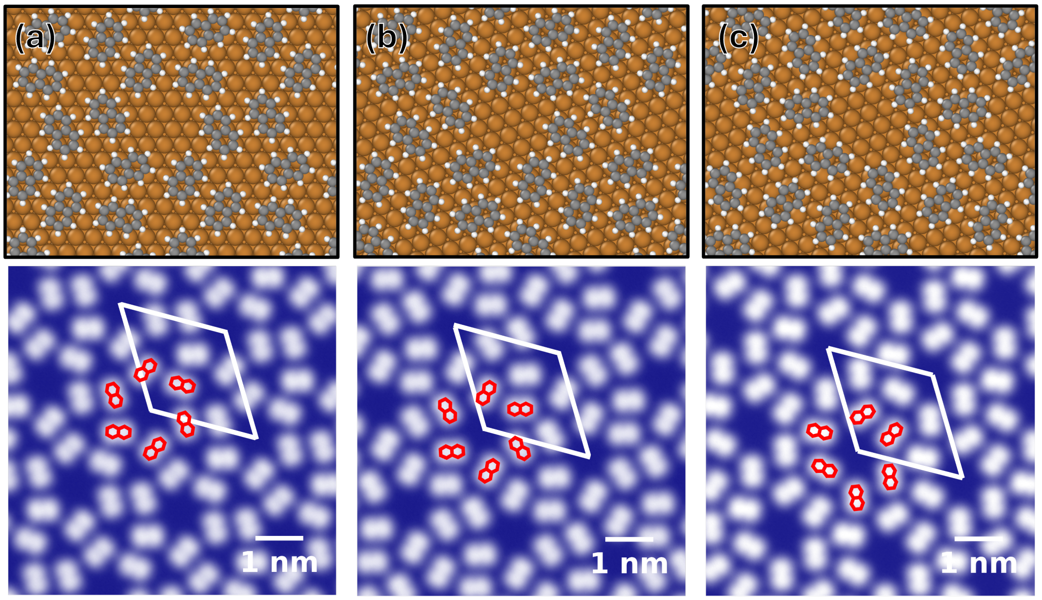


**Figure S20:** The three most stable structures of naphthalene on Cu(111) near the experimental coverage bin for Z = 6, marked with red dashed lines in Figure 17 in the main text. Panel a and b correspond to the structures circled in orange and green in Figure 17. Panel c shows the second most stable structure with the experimental coverage. The simulated STM image of each structure is shown below.

the naphthalene atoms is shown in purple. The main contributions originate from the Cu $d$ states, and the film states are located far away from the Fermi level. In the binding energy range between $-2\,eV$ to $0\,eV$, shown in the experimental UPS, no film states exist based on our calculations. therefore, it is not possible to deduce the structure of the naphthalene film based on DOS signatures. The UPS measurement shows a fermi level shift of 0.19 eV due to the adsorption of naphthalene, measured by the spacing between the first Cu DOS peaks as indicated by the black arrow in Fig. S21 (b). For the computed DOS, the Fermi level shift is estimated based on the shift of the Cu $d$-band peak in the region of -2.5 eV to -2 eV. The shift of 0.19 eV reported in Ref.[5] is best reproduced by the Z=1 rectangular structure, which matches the one observed at 120 K.

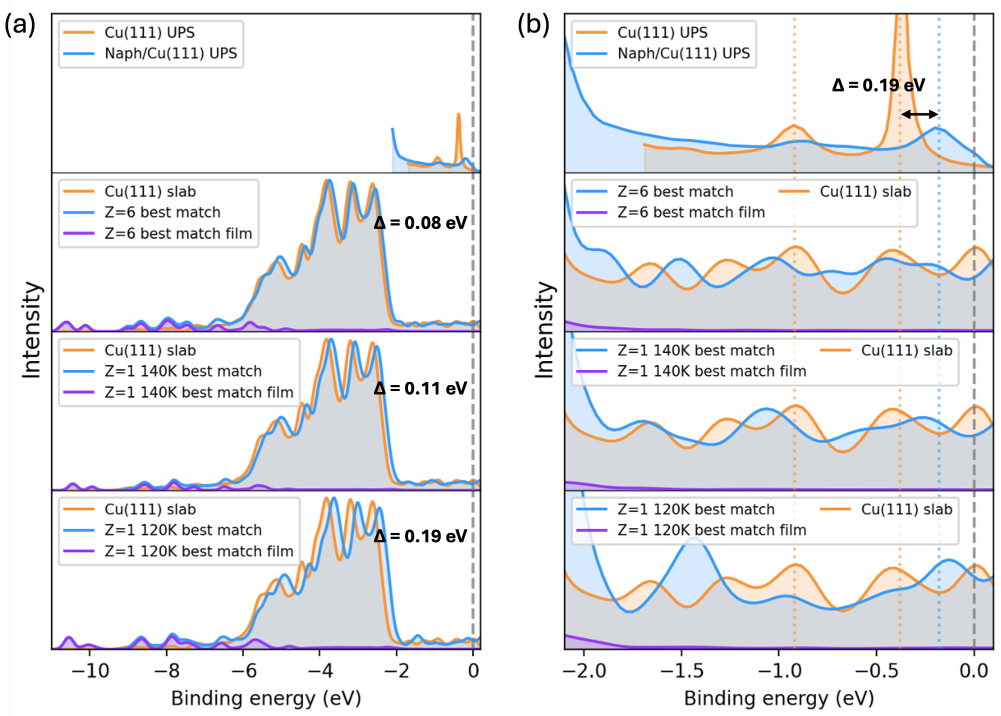


**Figure S21:** Calculated density of states of naphthalene/Cu(111) interface structures generated by Genarris. (a) The overall DOS from -11 eV to 0 eV. (b) Magnified view of the DOS from -2 eV to 0 eV. The top panel is the UPS data reproduced with permission from Ref.,[5] Copyright 2019 American Chemical Society. The Fermi level is indicated by the gray dashed line. The shift of the Fermi level Δ due to naphthalene adsorption is also shown.

# References


[1] Tersoff, J.; Hamann, D. R. *Physical Review B* **1985**, *31*, 805.

[2] Perdew, J. P.; Burke, K.; Wang, Y. *Phys. Rev. B* **1996**, *54*, 16533–16539.

[3] Ruiz, V. G.; Liu, W.; Zojer, E.; Scheffler, M.; Tkatchenko, A. *Phys. Rev. Lett.* **2012**, *108*, 146103.

[4] Obersteiner, V.; Scherbela, M.; Hormann, L.; Wegner, D.; Hofmann, O. T. *Nano Letters* **2017**, *17*, 4453–4460.

[5] Klein, B. P.; Morbec, J. M.; Franke, M.; Greulich, K. K.; Sachs, M.; Parhizkar, S.; Bocquet, F. C.; Schmid, M.; Hall, S. J.; Maurer, R. J.; Meyer, B.; Tonner, R.; Kumpf, C.; Kratzer, P.; Gottfried, J. M. *The Journal of Physical Chemistry C* **2019**, *123*, 29219–29230.
